# VTH BRAZILIAN CONSENSUS CONFERENCE ON *HELICOBACTER PYLOR*I INFECTION

**DOI:** 10.1590/S0004-2803.24612026-043

**Published:** 2026-08-10

**Authors:** Luiz Gonzaga Vaz COELHO, Bruno Squárcio Fernandes SANCHES, Ismael MAGUILNIK, James Ramalho MARINHO, Sandro Rodrigues CHAVES, Ana Flávia P. RAMOS, Heinrich SEIDLER, Ricardo C. BARBUTI, Renato S. LABOISSIÈRE, Laércio Tenório RIBEIRO, Maria do Carmo F. PASSOS, Julio César de Soares VELOSO, Lucia Libanês C. BRAGA, Frederico Passos MARINHO, Helenice P. BREYER, Hoiti OKAMOTO, Jardel Soares CAETANO, Paulo Pimentel D’ASSUMPÇÃO, Leonardo Soares da SILVA, Gerson DOMINGUES, Raul Carlos WAHLE, Eliza Maria de BRITO, Marcela Tavares da Rocha LOURES, Joaquim Prado MORAES-FILHO, Décio CHINZON, Áureo Almeida DELGADO

**Affiliations:** 1 Universidade Federal de Minas Gerais, MG, Brasil.; 2 Universidade Federal do Rio Grande do Sul, RS, Brasil.; 3 Universidade Estadual de Ciências da Saúde de Alagoas, AL, Brasil.; 4 Rede Mater Dei de Saúde, MG, Brasil.; 5 Santa Casa de Belo Horizonte, MG, Brasil.; 6 Universidade Católica de Brasília, DF, Brasil.; 7 Universidade de São Paulo, SP, Brasil.; 8 Hospital Universitário Prof. Alberto Antunes, AL, Brasil.; 9 Hospital Santa Helena, DF, Brasil.; 10 Universidade Federal do Ceará, CE, Brasil.; 11 Centro Médico Oxford, SC, Brasil.; 12 Hospital Cárdio Pulmonar (Rede D’Or São Luiz), BA, Brasil.; 13 Universidade Federal do Pará, PA, Brasil.; 14 Hospital Adventista de Manaus, AM, Brasil.; 15 Universidade do Estado do Rio de Janeiro, RJ, Brasil.; 16 Universidade Federal de São Paulo, SP, Brasil.; 17 Universidade Professor Edson Antônio Vellano, MG, Brasil.; 18 Hospital Nossa Senhora das Graças, PR, Brasil.; 19 Universidade Federal de Juiz de Fora, MG, Brasil.

**Keywords:** Helicobacter pylori, H. Pylori, Consensus, Helicobacter pylori, H. pylori, Consenso

## Abstract

**Background:**

Helicobacter pylori infection remains highly prevalent, affecting approximately 40% of the global population. Individuals with infection continue to be at risk for its various clinical consequences, including chronic gastritis, peptic ulcer disease, and gastric cancer.

**Objective:**

The Brazilian Helicobacter pylori and Microbiota Study Group and the Brazilian Federation of Gastroenterology present the Fifth Brazilian Consensus on this topic, following the inaugural edition in 1995. Significant advances across various areas have emerged since the publication of the last consensus in 2018. These advances are particularly relevant to bacterial eradication, as treatment encounters mounting challenges due to rising antimicrobial resistance.

**Methods:**

For this update, five working groups were established to address: 1) epidemiology; 2) associated diseases and treatment indications; 3) diagnosis; 4) gastric cancer; and 5) treatment and microbiota.

**Conclusion:**

The groups developed 34 evidence-based recommendations to guide clinicians in managing this infection within the Brazilian population.

## HIGHLIGHTS

• The consensus brought together 26 experts from all regions of Brazil, who developed 34 evidence-based recommendations to guide infection management in Brazil population.

• Removing the bacterium is recommended for all infected individuals and is crucial in cases of chronic gastritis, peptic ulcer disease, MALT lymphoma, and gastric precancerous conditions.

• The ¹³C-urea breath test is the non-invasive gold standard for diagnosis and treatment monitoring, while histology is the invasive gold standard for mucosal assessment.

• In response to increasing antimicrobial resistance, the consensus recommends 14-day treatments, emphasizing quadruple bismuth therapy and the use of P-CABs (such as vonoprazan) to maximize effectiveness.

• Eradication of *H. pylori* is crucial for the primary and secondary prevention of gastric cancer, particularly among first-degree relatives of patients with the disease and following resection of early tumors.

## Resumo

**Conexto:**

A infecção por *Helicobacter pylori* é ainda muito frequente e acomete aproximadamente 40% da população mundial, que segue exposta a suas diferentes consequências clínicas como gastrite crônica, úlcera péptica e câncer gástrico, entre outras.

**Objetivo:**

O Núcleo Brasileiro para Estudo do Helicobacter pylori e Microbiota e a Federação Brasileira da Gastroenterologia promovem agora seu V Consenso Brasileiro sobre o tema, cuja primeira edição ocorreu em 1995. Importantes avanços, em diferentes tópicos, foram observados desde a publicação de seu último consenso em 2018, especialmente no tocante a erradicação da bactéria, cujo tratamento enfrenta dificuldades crescentes pelo aumento da resistência antimicrobiana.

**Métodos:**

Para esta atualização foram criados cinco grupos de trabalhos abordando: 1) epidemiologia; 2) doenças associadas e indicações de tratamento; 3) diagnóstico; 4) câncer gástrico e, 5) tratamento e microbiota.

**Conclusão:**

Foram elaboradas 34 recomendações fundamentadas nas melhores evidências para auxiliar os médicos no manuseio desta infecção na população brasileira.

## INTRODUCTION

*Helicobacter pylori* (*H. pylori*) infection affects the stomach of over 40% of the global population and can cause a wide spectrum of digestive diseases, such as chronic gastritis, peptic ulcer disease, gastric adenocarcinoma, and gastric MALT lymphoma^1^. To date, four consensus meetings on its management have been held in Brazil, namely in 1995^2^, 2004^3^, 2012^4^, and 2018^5^. The global rise in resistance to antibiotics commonly used in eradication therapy (e.g., clarithromycin, levofloxacin, and metronidazole) reduces expected treatment benefits. Coupled with the lack of new antimicrobials targeting this bacterium^6^, these factors raise growing concerns regarding infection management. However, significant developments have occurred in novel therapeutic regimens that use potent antisecretory agents combined with new dosing schedules, necessitating a new national consensus. Furthermore, despite the limited phenotypic and genotypic studies evaluating *H. pylori* characteristics in the Brazilian setting^7^ ^,^ ^8^, initial results are now available from well-structured, real-world studies encompassing all Brazilian regions. These data were obtained through the Brazilian Registry on *H. pylori* Management (Hp-BraReg), created in 2021 in partnership with the European Registry on *H. pylori* Management (Hp-EuReg), which together now constitute the World Registry on *H. pylori* Management (WorldHpReg)^9^ ^,^ ^10^. Although the Kyoto Consensus^11^ established that every individual with an infection should be treated, the primary treatment indications have been expanded and detailed with new recommendations. Various diagnostic advances are discussed, including non-invasive, endoscopic, and histopathological methods, alongside an arsenal of molecular tests specifically designed to detect antimicrobial resistance. These advances aim to introduce an as-yet elusive antimicrobial stewardship policy into routine clinical practice. The growing role of the gastric and intestinal microbiota and the potential use of probiotics during treatment are discussed, considering current knowledge, along with different treatment strategies for primary and secondary gastric cancer prevention. Accordingly, the Brazilian *Helicobacter pylori* and Microbiota Study Group (Núcleo Brasileiro para Estudo do Helicobacter pylori e Microbiota; NBEHPM, per its Portuguese acronym), supported by the Brazilian Federation of Gastroenterology (Federação Brasileira de Gastroenterologia; FBG), held its fifth meeting in Bento Gonçalves, Rio Grande do Sul, from October 9 to 11, 2025. A total of 26 panelists-including gastroenterologists, endoscopists, and pathologists from all five of the country’s regions-participated in the meeting. Participants were selected based on their expertise and contributions to the field.

## Consensus Development Process

The Organizing Committee adopted the Delphi method^12^. A total of 34 clinical questions were selected and divided among five working groups focused on 1) epidemiology; 2) associated diseases and treatment indications; 3) diagnosis; 4) gastric cancer; and 5) treatment and microbiota. The 26 delegates were assigned to these groups based on their expertise, with one member from each group invited to serve as a moderator. Each delegate prepared one or two draft statements with references pertaining to up to two clinical questions, delivering oral presentations along with their recommendations during two separate virtual meetings restricted to their respective group. The draft statements and recommendations were discussed, modified when necessary, and edited electronically during the meetings by FBG staff, followed by anonymous voting. Subsequently, in a third meeting-now an in-person plenary session in Bento Gonçalves with all participants present-all recommendations were voted on again, re-discussed, and modified as needed. Voting allowed for five options: 1) strongly agree; 2) agree with reservations; 3) undecided; 4) disagree; and 5) strongly disagree. Decisions were based on the risk of bias in the studies, evidence of publication bias, heterogeneity among studies, indirectness of the evidence, and precision of the effect estimate.

The consensus threshold for the final approval of each recommendation was 80% of voters who responded with either “strongly agree” or “agree with reservations”. The level of evidence and the strength of the recommendations were classified using the GRADE system (Grading of Recommendations Assessment, Development and Evaluation^13^ ^-^ ^15^), summarized below:

## Quality of Evidence

- High quality Further research is very unlikely to change our confidence in the estimate of effect.
- Moderate quality Further research is likely to have an important impact on our confidence in the estimate of effect and may change the estimate.
- Low quality Further research is very likely to have an important impact on our confidence in the estimate of effect and is likely to change the estimate.
- Very low quality.

Any estimate of effect is very uncertain.

The strength of the recommendation was classified as strong (recommendations) or weak (suggestions), depending on the quality of evidence, cost-benefit ratio, feasibility, and costs.

## Strength of Recommendation

1. Strong recommendation Most patients should receive the recommended intervention.
2. Weak recommendation.

Clinicians should recognize that different choices are appropriate for different patients, and they must help each patient arrive at a management decision consistent with the patient’s values and preferences.

## Working group 1: epidemiology

### Statement 1

### The prevalence of *H. pylori* is primarily related to socioeconomic and hygiene conditions, as well as cultural habits. Transmission within the family is possible.

**Agreement: 100%**

**GRADE: 1A | Strength of recommendation: strong**

**Quality of evidence: high**

Two large systematic reviews and meta-analyses have evaluated the global prevalence of *H. pylori* infection over the past decades^1^ ^,^ ^16^. Li Y et al. assessed infection prevalence between 1980 and 2022 across 224 studies from 71 countries involving nearly 3,000,000 individuals. They estimated a reduction in infection rates from 58.2% in the 1980s to 43.1% in the 2011-2022 period. Infection prevalence shows marked geographical heterogeneity, with a higher infection load observed in developing countries and rural areas compared to urban or developed regions^1^. Another large study assessing *H. pylori* prevalence and gastric cancer incidence across 1,748 studies confirmed this trend, showing a reduction in prevalence from 52.6% in adults before 1990 to 43.9% in the 2015-2022 period. The study estimated a 15.9% reduction over the past 3 decades in adults, though the decline was less significant in children, where prevalence rates remained as high as 35.1%^16^. As with most endemic infectious diseases, a decline in prevalence is more closely tied to improvements in population hygiene and sanitation than to individualized case-by-case treatment since, in most countries (except Japan, for example), only a minority of individuals with infection receive treatment^17^. Over 40% of the world’s population continues to carry the infection and remains at risk for potential clinical sequelae. The Brazilian studies included in these two systematic reviews, along with recent studies, confirm this downward trend in adult prevalence, even when accounting for regional socioeconomic and sanitary variations^18^ ^,^ ^19^. A cross-sectional study using the ^13^C-urea breath test as a diagnostic tool in 161 children (mean age 7.8 years) in a southeastern Brazilian city found an *H. pylori* prevalence of 20.5%^20^.

*H. pylori* infection is largely considered a familial disease. Person-to-person transmission within families, especially from mothers and siblings with the infection, is common in developing countries^21^. Genotyping studies have shown that strain concordance was detected in 10 of 18 (56%) mother-child pairs and in zero of 17 father-child pairs. Concordant strains were also observed among siblings in 29 of 36 (81%) families^22^. However, transmission between couples or spouses remains controversial^23^ ^,^ ^24^. Large-scale Chinese epidemiological studies based on family-level treatment indicate this strategy contains intrafamilial infection spread in highly endemic areas^25^ ^,^ ^26^.

### Statement 2

### Risk groups for *H. pylori* infection include populations with poor basic sanitation, rural communities, households with documented cases, healthcare professionals, and patients who are immunocompromised or those with comorbidities. *H. pylori* transmission occurs primarily in childhood, with the fecal-oral route being the most relevant.

**Agreement: 100%**

**GRADE: 1A | Strength of recommendation: strong**

**Quality of evidence: high**

Sanitary, socioeconomic, and environmental factors represent the primary determinants for acquiring *H. pylori* infection^27^. However, other population groups and clinical conditions also convey an elevated risk for acquiring and/or harboring the infection. Childhood is considered the critical period for acquisition, with most cases occurring before 10 years of age, likely due to greater exposure in environments with poor hygiene and closer interpersonal contact^28^. In older adults, the lifetime cumulative prevalence is associated with an increased risk of complications, such as atrophic gastritis, intestinal metaplasia, and gastric cancer^29^. Transmission patterns are predominantly person-to-person, although foodborne, waterborne, animal-to-human, and occupational pathways are well-documented^30^ ^-^ ^35^. Person-to-person transmission within households, particularly from mothers with the infection and siblings, is common in developing countries^30^. There are several transmission routes for *H. pylori*, with the fecal-oral and oral-oral routes being the most likely. Other described routes include gastro-oral, anal-oral, and genital-oral^31^ ^,^ ^33^ ^,^ ^34^. Nonetheless, further studies on transmission pathways and their relative importance are still needed^30^. Documented household infection, especially in the mother-child pattern, represents a significant transmission route, and family screening has proven beneficial in preventing associated complications^25^ ^,^ ^26^. Certain health conditions also confer increased vulnerability. Patients who are immunocompromised, such as those undergoing chemotherapy, transplant recipients, or people living with HIV, demonstrate heightened susceptibility and impaired infection control^36^. Furthermore, patients with chronic diseases, such as diabetes and cardiovascular conditions, appear to have a higher likelihood of acquiring the infection, though this remains controversial^37^. Racial and ethnic disparities have also been documented in the United States, where rates are higher among Black and Hispanic individuals than among White individuals. These differences may be attributed to both genetic factors and unfavorable socioeconomic and environmental conditions^38^. While the occupational risk remains controversial, a systematic review of 98 studies suggested that healthcare professionals, especially those in the gastrointestinal field, face a higher risk, indicating an iatrogenic transmission route^39^. The detection of *H. pylori* DNA in vomit, saliva, dental plaque, gastric juice, and stool supports its potential transmission via endoscopic procedures^40^ ^-^ ^43^. A recent Brazilian study found no differences in infection prevalence between clinical gastroenterologists and GI endoscopists, highlighting the effectiveness of current universal disinfection and protection protocols^18^.

### Statement 3

### Recurrence of *H. pylori* infection in adults is low in developed countries, with reinfection being more frequent in regions with higher prevalence.

**Agreement: 88%**

**GRADE: 1A | Strength of recommendation: strong**

**Quality of evidence: high**

Based on clinical and temporal criteria, two types of *H. pylori* infection recurrence after eradication therapy can be identified^44^ ^,^ ^45^:

**Recrudescence -** A relapse occurring within one year post-treatment. This process involves the resurgence of the original strain that was temporarily suppressed; thus, the bacterium responsible for the recurrence is genetically identical to the strain identified prior to eradication therapy.

**Reinfection -** Defined as an infection by a new *H. pylori* strain following successful eradication. Some authors posit a temporal criterion, suggesting reinfection is responsible when a diagnostic test yields a positive result at least 1 year after the initial eradication^44^ ^,^ ^45^.

Definitive confirmation, however, is achievable only through molecular analysis comparing strains isolated before and after relapse, a capability practically unavailable in routine clinical practice. Recrudescence is more common when low-efficacy therapies are utilized. Reinfection requires re-exposure and is therefore more likely in countries with high *H. pylori* prevalence and poor sanitation^44^. The annual rate of reinfection or recrudescence following successful eradication is low (<2%) among adults in developed countries, but higher (5-10%) among adults in developing countries and among children^30^. Several randomized clinical trials have demonstrated that family-based *H. pylori* screen-and-treat strategies reduce recurrence rates more effectively than treating individuals alone^30^. A systematic review and meta-analysis encompassing 31 studies (16,797 participants) revealed a pooled *H. pylori* recurrence rate of 9% using a random-effects model, showing an upward trend as time elapsed since eradication^46^. At 1 year of follow-up, the *H. pylori* recurrence rate was 4%; at 2 years, 6%; at 3 years, 8%; and at 4 years or beyond, it reached 12%, eventually exhibiting a stabilization trend. The *H. pylori* recurrence rate was inversely related to the Human Development Index.

Brazilian studies on this topic are scarce. A study conducted in the state of Minas Gerais analyzed 150 patients with duodenal ulcers who achieved successful *H. pylori* eradication and were followed for a median period of 6.4 years (range: 305 days to 8.9 years). Twenty of the 150 patients (13.3%) reverted to a positive result on the ^14^C-urea breath test, yielding an annual recurrence rate of 2.1%^47^. In the state of São Paulo, two studies were conducted: one identified a recurrence rate of 5.7% while following 194 patients over a 10-year period^48^. Another study involving 147 patients demonstrated an annual recurrence rate of 1.8% over a 5-year follow-up, with no recurrence at 1 year^49^.

## Working group 2: associated diseases and treatment indications

Table 1 shows the main indications of *H. pylori* treatment.

**TABLE 1:** Indications of *H. pylori* treatment.

Chronic gastritis
Peptic ulcer disease
Functional dyspepsia/H. pylori-associated dyspepsia
Uninvestigated dyspepsia in patients under 45 years of age without alarm symptoms.
Chronic gastritis and associated pre-neoplasic conditions or lesions (atrophy, intestinal metaplasia and dysplasia)
Gastric MALT (mucosa-associated lymphoid tissue) lymphoma
Post-endoscopic or surgical resection of early gastric cancer
Family members (parents, siblings, and children) of gastric cancer patients
Planned long-term treatment with non-steroidal antiinflammatory drugs including aspirin
Patients with gastroesophageal reflux disease with recommendations for periodic use of PPIs or P-CABs
Unexplained iron deficiency anaemia or immune thrombocytopenic purpura
Bariatric surgery candidates
Patient´s request

PPI: proton pum inhibitor. P-CAB: potassium-competitive acid blocker.

### Statement 4

### Eradication therapy is recommended for all cases of *H. pylori*-associated chronic gastritis.

**Agreement: 96%**

**GRADE: 1A | Strength of recommendation: strong**

**Quality of evidence: high**

*H. pylori* infection is considered the leading cause of chronic gastritis, which drives persistent inflammation of the gastric mucosa, with potential progression to tissue damage and an elevated risk for severe complications such as peptic ulcer disease, gastric adenocarcinoma, and gastric MALT lymphoma^11^ ^,^ ^50^. For most patients, apparent clinical symptoms are absent despite structural and functional abnormalities resulting from mucosal inflammation^50^ ^,^ ^51^. The interaction among different *H. pylori* genotypes, the duration of infection, host genetics, and environmental factors leads to distinct outcomes and complications^52^.

*H. pylori* infection universally causes gastric mucosal inflammation, characterized by neutrophilic and lymphoplasmacytic infiltrates. The combination of neutrophil-mediated injury and a cytotoxic immune response-often induced by cagA+ strain polymorphisms-can lead to loss of tissue integrity (ulceration) and glandular depletion (atrophy). This atrophy, in turn, establishes the groundwork for gastric cancer development^53^.

While *H. pylori* infection is the leading cause of chronic gastritis, other causes encompass communicable (infectious) conditions, non-communicable host-related conditions such as autoimmune diseases (autoimmune gastritis), and immune-mediated conditions (e.g., Crohn’s disease), and drug-induced injuries (e.g., immunotherapy)^11^. Brazil presents a broad epidemiological spectrum of *H. pylori* gastritis, varying both regionally (and micro-regionally) and by age group, reflecting disparities in population access to basic sanitation and eradication therapy. Consequently, the etiological diagnosis of gastritis must be established to ensure an appropriate therapeutic approach.

Histology is a simple, cost-effective, and widely available test that facilitates *H. pylori* identification and structural assessment of the mucosa. The density of *H. pylori* distribution across the gastric mucosa is variable and influenced by anatomical site, atrophy/metaplasia, and medication use. Therefore, when an upper GI endoscopy is indicated, systematic tissue sampling following established protocols is recommended. The updated Sydney System recommends biopsies from the lesser and greater curvatures of the antrum and corpus (four samples) and a fifth from the incisura angularis^54^. More recently, the MAPS II guidelines recommend two biopsies from the antrum and two from the corpus^55^. Areas with an atrophic appearance and suspicious lesions warrant targeted biopsies. Samples must be placed in separate vials by site and properly identified. *H. pylori* is easily identified using hematoxylin and eosin (H&E) staining. Special staining techniques (such as Giemsa) and immunohistochemistry increase the probability of detection but entail higher costs and longer diagnostic turnaround times. They are preferentially indicated in special situations, such as MALT lymphoma, active chronic gastritis, and chronic atrophic gastritis, where infection is suspected, but the bacterium remains unidentified by H&E^56^. The histology report must include the gastritis etiology (probable or suspected), the intensity of inflammation, and the staging of atrophy and metaplasia.

*H. pylori* eradication eliminates inflammatory activity, halts the progression of tissue damage, and reduces the risk of future complications. Furthermore, it improves the control of dyspeptic symptoms and reduces the risk of bacterial transmission. Thus, eradication therapy is indicated for all patients with *H. pylori*-associated chronic gastritis^51^.

### Statement 5

### *H. pylori* eradication is recommended in patients with gastric or duodenal peptic ulcers, as long as therapeutic options remain available.

**Agreement: 100%**

**GRADE: 1A | Strength of recommendation: strong**

**Quality of evidence: high**

Multiple studies demonstrate a declining prevalence of *H. pylori*-associated peptic ulcer disease in recent decades^57^ ^-^ ^59^. This decline is aligned with the overall global reduction in *H. pylori* prevalence^1^, population-based treatment in some countries, and improved living conditions, despite the concurrent upward trend in ulcers associated with the use of aspirin and NSAIDs^59^. Despite the decreasing prevalence trend, *H. pylori* remains the leading cause of gastric or duodenal ulcers. A population-based prospective cohort study comprising 2416 adults from Denmark showed an odds ratio of 4.3 (95%CI 2.2-8.3) for the association between *H. pylori* infection and gastric or duodenal peptic ulcers^60^. In a prospective German study, the risk of developing a duodenal ulcer and a gastric ulcer was 18.4 and 2.9 times higher, respectively, among individuals with cagA+ *H. pylori* infections^61^. A recent Korean multicenter study involving 26,785 individuals (38.8% over age 65 years) described the main risk factors for peptic ulcer disease as *H. pylori* infection (41.8%), use of aspirin or NSAIDs (36.1%), and undetermined causes in 22.1% of cases^62^.

*H. pylori* infection promotes two distinct types of gastritis. The first involves the gastric antrum (antrum-predominant gastritis) and causes acid hypersecretion by interfering with acid regulatory pathways^63^. Increased acid secretion delivers a higher acid load to the duodenum, elevating duodenal susceptibility by inducing the development (or extension, if already present) of gastric metaplasia areas in the duodenum. This allows *H. pylori* colonization, rendering the duodenal epithelium more vulnerable and prone to ulceration. The second gastritis pattern associated with *H. pylori* involves the entire stomach (pangastritis) and is linked to gastric atrophy, reduced acid secretion, and gastric cancer. *H. pylori* is considered responsible for 70% to 80% of benign gastric ulcerations. The lower prevalence of the bacterium in gastric ulcers compared to duodenal ulcers is related to the higher frequency of NSAID-induced ulcers. Gastric ulcers tend to occur in non-acid-secreting mucosa or near the junction with non-secreting mucosa. Even when they occur high on the lesser curvature, they arise in non-secreting mucosa. Under these circumstances, *H. pylori*-induced pangastritis drives the metaplastic changes that convert secreting mucosa into non-secreting mucosa. Refluxed duodenal contents, especially bile and lecithin, damage the gastric mucosa, making it more sensitive to acid-induced injury, even in small amounts. Furthermore, *H. pylori* infection can alter the mucin layer coating the gastric epithelium and compromise mucosal defense mechanisms^64^ ^,^ ^65^.

*H. pylori* eradication promotes ulcer healing, reduces the risk of recurrence, and prevents severe complications such as bleeding and gastric cancer, rendering it a universal recommendation^5^ ^,^ ^11^ ^,^ ^17^ ^,^ ^38^ ^,^ ^51^. Among patients with successful eradication, the annual ulcer recurrence rate is approximately 0% to 2.3% for gastric ulcers and 0% to 1.6% for duodenal ulcers during a follow-up period of up to 9.8 years^66^ ^,^ ^67^. In contrast, patients with untreated *H. pylori* infections faced recurrence rates of 52% for gastric ulcers and 64% for duodenal ulcers^68^.

### Statement 6

### The “test-and-treat” strategy is recommended for patients aged 45 years or younger with uninvestigated dyspepsia and no alarm features.

**Agreement: 95%**

**GRADE: 1A | Strength of recommendation: strong**

**Quality of evidence: high**

Dyspepsia represents a common clinical condition with an estimated prevalence of approximately 20% in the general population^69^. It is characterized by pain or discomfort in the upper gastrointestinal tract (the gastroduodenal region) and classified as investigated or uninvestigated^70^. According to the Rome IV consensus, after investigation via upper GI endoscopy, dyspepsia can be classified as^70^:

1. Organic: when symptoms are explained by endoscopic findings, such as an ulcer or gastric cancer.
2. *H. pylori*-associated: when infection is present, and *H. pylori* eradication leads to sustained resolution of dyspeptic symptoms for over 6 months.
3. Functional: when relevant endoscopic abnormalities or *H. pylori* infection are absent, or, if infected, symptoms persist even after eradication therapy. This category comprises the vast majority of dyspepsia cases.

For individuals aged 45 years or younger presenting with dyspepsia without alarm features, management strategies have been evaluated in clinical trials and meta-analyses. Approaches include empirical treatment with proton pump inhibitors (PPIs), immediate endoscopy, the “test-and-treat” strategy for *H. pylori*, or testing followed by endoscopy if positive. The “test-and-treat” strategy has proved to be the most effective and cost-effective approach, particularly in regions with a high prevalence of infection, such as Brazil^51^ ^,^ ^71^ ^-^ ^78^.

A systematic review and network meta-analysis by Eusebi et al. evaluated 15 randomized controlled trials with 6,162 patients, comparing all strategies directly and indirectly. “Test-and-treat” ranked highest for symptom reduction, in both intention-to-treat and per-protocol analyses. Immediate endoscopy performed similarly, but the “test-and-treat” strategy led to a significantly lower number of endoscopies during the 12-month follow-up, offering substantial cost-saving benefits. The neoplasia detection rate was very low (0.4%) across all treatment arms^78^. Additional benefits of the “test-and-treat” strategy for uninvestigated dyspepsia include a reduction in the future incidence of peptic ulcer disease and gastric cancer, as well as reduced management costs for these conditions^79^.

### Statement 7

### *H. pylori* eradication therapy is recommended as the first-line therapeutic option for patients with investigated dyspepsia and provides a modest but significant benefit for symptom improvement. Symptom persistence or relapse despite eradication characterizes functional dyspepsia.

**Agreement: 93%**

**GRADE: 1A | Strength of recommendation: strong**

**Quality of evidence: high**

Functional dyspepsia, defined by the Rome IV consensus as a clinical syndrome characterized by recurrent and chronic dyspeptic symptoms without underlying structural or metabolic lesions to explain them, accounts for nearly 80% of dyspepsia cases^70^. While the condition does not impact life expectancy, it is a chronic disorder with a fluctuating course that profoundly impacts patients’ quality of life and carries a high financial burden^70^ ^,^ ^80^. Its pathophysiology is complex and multifactorial, with several proposed mechanisms, including alterations in the gastroduodenal microbiota^70^.

Among patients with dyspepsia, once *H. pylori* infection is confirmed, eradication treatment should be the first therapy instituted. Recent high-quality meta-analyses, systematic reviews, and randomized clinical trials have consistently shown that *H. pylori* eradication leads to a modest but statistically significant improvement in dyspepsia symptoms. The Number Needed to Treat (NNT) ranges from 9 to 15 for symptom improvement and from 11 to 21 for a cure^81^ ^-^ ^83^. The treatment benefit is most pronounced among patients with successful eradication^81^.

However, sustained symptom resolution following eradication characterizes the condition as *H. pylori*-associated dyspepsia, rather than functional dyspepsia, per Rome IV criteria^70^. Selecting initial *H. pylori* eradication in functional dyspepsia, particularly in highly endemic regions, is also advocated by major international consensus guidelines due to its cost-effectiveness^11^ ^,^ ^51^ ^,^ ^84^. One consideration is that eradication treatment carries a higher rate of side effects, though severe adverse events are rare^82^ ^,^ ^83^. Unanswered questions persist in this area, including whether symptomatic improvement results from gastroduodenal microbiota modulation by antibiotics, which patient subgroups benefit the most, what the long-term impact on quality of life entails, and what the true role of genetic and regional factors involves.

### Statement 8

### *H. pylori* eradication is recommended in all cases of precancerous conditions (atrophic gastritis and intestinal metaplasia) and precancerous lesions (dysplasia) of the gastric mucosa, given the likelihood of reducing gastric carcinoma risk.

**Agreement: 100%**

**GRADE: 1A | Strength of recommendation: strong**

**Quality of evidence: high**

The progression of normal mucosa toward non-hereditary gastric carcinoma (GC) involves multiple stages, starting with chronic gastritis. Prolonged, non-self-limiting inflammation in response to persistent *H. pylori* infection, as well as the direct action of the bacterium itself, facilitates adaptive responses such as atrophy and metaplasia. In a smaller subset of patients-especially individuals with a positive family history, smoking habits, and high dietary sodium-the mutation rate is higher in the metaplastic epithelium, from which dysplasia and GC, predominantly of the intestinal type, arise^50^. Gastric atrophy (GA) is characterized by glandular loss and may or may not be associated with gastric intestinal metaplasia (GIM), defined by the replacement of the foveolar epithelium with cells exhibiting an intestinal phenotype. GIM can be subdivided into complete and incomplete, reflecting small intestine and colonic phenotypes, respectively. Absorptive cells with a brush border and Paneth cells define the complete type, whereas non-absorptive columnar cells, alongside goblet cells, characterize the incomplete pattern, which conveys a higher risk of malignant transformation and is often seen in extensive GIM cases^50^ ^,^ ^85^. There is also a GIM classification based on mucin histochemical profiling, subtyping it into I, II, and III, with type III harboring the highest carcinogenic potential^86^. However, due to reagent toxicity, this classification is not recommended in routine practice^87^.

Dysplasia is phenotypically characterized by reduced mucosecretory differentiation and increased cell proliferation, displaying cytologic/architectural atypia without signs of stromal invasion. Based on its complexity, it is classified as low-grade and high-grade, conveying a progressive cancer risk. When the histopathological picture cannot be distinguished from regenerative changes, the case is deemed indefinite for dysplasia. A repeat biopsy is recommended, as only 9% of cases remain indefinite upon a second biopsy^88^ ^,^ ^89^.

GA and GIM progressively increase the risk for GC but possess the potential for regression following *H. pylori* treatment, defining these changes as precancerous conditions^50^ ^,^ ^90^. Notably, the likelihood of regression drops significantly when these conditions extensively involve the corpus and antrum^91^. In contrast, dysplasia is designated as a precancerous lesion, as it exhibits a higher risk of evolving into invasive GC and a lower chance of regression following antibiotic therapy^50^ ^,^ ^87^.

Long-term studies demonstrate that *H. pylori* eradication in patients with GA and GIM leads to significant regression of these conditions and reduces their progression to more advanced stages^92^ ^-^ ^94^. An enhanced benefit is anticipated when treatment occurs in the early stages. However, long-term improvement can occur even with extensive GA/GIM, when endoscopic surveillance is indicated^90^ (see recommendation 22). For precancerous lesions, the chance of regression is lower, even in low-grade dysplasia, but eradication still reduces the GC risk, even in high-grade dysplasia cases^94^ ^,^ ^95^. It is recommended to test for *H. pylori* using non-invasive methods in extensive GA/GIM, as the bacterial population is typically reduced in these settings, hindering its detection in histological sections.

### Statement 9

### Patients with gastric MALT lymphoma and *H. pylori* infection must receive eradication treatment. *H. pylori*-negative patients may benefit from empirical treatment.

**Agreement: 100%**

**GRADE: 1A | Strength of recommendation: strong**

**Quality of evidence: high**

MALT lymphoma is an extranodal low-grade B-cell lymphoma characterized by the clonal expansion of marginal zone cells of mucosa-associated lymphoid tissue (MALT). As a neoplasm that reflects the behavioral, cytoarchitectural, and immunophenotypic features of normal MALT^96^, it accounts for 30-60% of primary gastric lymphomas^97^. In addition, its development is strongly linked to *H. pylori* infection, which is present in up to 90% of cases^98^. Recent studies indicate a decreasing incidence of gastric MALT lymphoma and a lower proportion of cases associated with *H. pylori* ^99^.

Testing for *H. pylori* infection must accompany the neoplasm diagnosis. Therefore, in addition to sampling the suspected neoplastic area, systematic gastric mucosa sampling following established protocols is recommended (see section on chronic gastritis). If H&E staining cannot identify the bacterium, infection must be ruled out by another method, including immunohistochemistry, urea breath test, or stool antigen testing.

Diagnosis of the disease must follow the current WHO classification^96^ and requires adequate tissue sampling for histological evaluation^100^ ^,^ ^101^. Histological diagnosis involves characterizing cellular composition, architecture, and identifying lymphoepithelial lesions^96^. While there is no single immunohistochemical marker, this evaluation allows for the immunophenotypic determination of cell composition, exclusion of other small cell lymphomas (mantle cell and follicular), better architectural characterization, and identification of lymphoepithelial lesions. The immunophenotype resembles that of marginal zone B-cells: CD20+, BCL2+, BCL6-, Cyclin D1-, CD5-, CD10-^96^.

*H. pylori* eradication therapy should be administered to all patients with *H. pylori*-positive MALT lymphoma, regardless of disease stage^102^ ^,^ ^103^. Treatment outcomes must be verified via breath testing or stool antigen testing at least 6 weeks after initiating therapy, at least 2 weeks after stopping PPIs, and 4 weeks after discontinuing antibiotics^102^. Histology during patient follow-up aims to assess the lymphoma’s histological response, graded by the GELA system, with the initial evaluation occurring 3 to 6 months post-eradication^104^. Complete Remission (CR) or Probable Minimal Residual Disease (pMRD) is considered a complete response, and regular endoscopic surveillance is recommended to ensure long-term lymphoma control. Responding Residual Disease (rRD) or No Change (NC) indicates persistent disease; when accompanied by symptomatic disease or signs of progression, specific treatment is required. For patients with clinical and endoscopic remission but microscopic lymphoma, it is reasonable to wait at least 12 months before initiating another treatment^102^.

Among *H. pylori*-negative cases, lymphoma regression following antibiotic treatment is less likely, and immediate initiation of specific anti-lymphoma treatments warrants consideration. Nevertheless, an empirical anti-*H. pylori* therapy trial can be justified, since a significant proportion of patients respond to treatment-possibly due to false-negative testing, infection by other *Helicobacter* species, or a direct antineoplastic effect^105^ ^,^ ^106^. Specific therapy should be considered if no endoscopic/histological response occurs 3 to 6 months post-eradication therapy^102^. For *H. pylori*-negative cases or those refractory to eradication, evaluation for chromosomal translocations (t^11;18^) via FISH is recommended^107^.

### Statement 10

### *H. pylori* eradication is recommended in all infected patients following endoscopic or surgical resection of gastric cancer.

**Agreement: 100%**

**GRADE: 1A | Strength of recommendation: strong**

**Quality of evidence: high**

For patients with a history of gastric cancer (GC), the residual stomach was potentially exposed to molecular driver events that promote carcinogenesis without necessarily developing all the alterations required for GC occurrence. However, this creates a high-risk environment for a new cancer. The field cancerization theory posits that carcinogenic injuries are not restricted to tumor cells but also affect areas adjacent to the cancer. Thus, even in the absence of identifiable histological lesions, molecular changes that increase cancer risk may be present in the residual stomach^108^ ^-^ ^111^. In this environment, hypothetically, eliminating the *H. pylori* infection reduces cancer risk by interrupting the cascade of events necessary for carcinogenesis. Particularly because of its interactions with other molecular driver mechanisms-such as DNA replication errors, microbiota shifts, and germline conditions that potentially affect the residual stomach-eradicating *H. pylori* lowers GC risk^108^. In cases of endoscopically resected early GC, the stomach is preserved and retains a microenvironment susceptible to persistent infection, and thus, to the risk of a new GC^109^.

Among post-gastrectomy patients, the microenvironment undergoes alterations and becomes less conducive to persistent infection; however, this increases susceptibility to other carcinogenic mechanisms, such as biliary reflux, elevated pH, and microbiota modifications. Additionally, diagnosing and treating *H. pylori* is more challenging in this new environment. Nonetheless, there is robust theoretical support for eradication to eliminate an additional risk factor^108^ ^,^ ^112^.

These molecular mechanisms are corroborated by clinical studies recommending *H. pylori* eradication in both settings. Following the endoscopic resection of early GC, randomized clinical trials and meta-analyses demonstrate a significant reduction in the incidence of metachronous neoplasms^113^ ^,^ ^114^. In 2025, Ford et al.^115^ evaluated three randomized clinical trials and two observational studies in patients undergoing endoscopic mucosal resection, showing that *H. pylori* eradication significantly lowers the risk of subsequent gastric cancer for these patients. These data bolster the strong recommendation, supported by high-quality evidence under GRADE methodology, for eradicating *H. pylori* after the endoscopic resection of early gastric cancer.

Conversely, patients undergoing surgical resection are evaluated primarily through observational studies and prospective cohorts. These studies indicate an association between *H. pylori* persistence and chronic inflammation of the residual mucosa, intestinal metaplasia, and the development of metachronous cancer^112^ ^,^ ^116^ ^,^ ^117^. Despite the lack of randomized trials in this scenario, the data align with established pathophysiological mechanisms, justifying a conditional recommendation with moderate-quality evidence.

### Statement 11

### First-degree relatives (parents, siblings, and children) of patients with gastric cancer should undergo testing and receive treatment if positive for *H. pylori* infection. Individuals aged 20 to 29 years should undergo bacterial testing using non-invasive methods, when available. Individuals aged 30 years and older should undergo an upper GI endoscopy with biopsies of the antrum and corpus to diagnose the infection and achieve adequate staging for follow-up.

**Agreement: 92%**

**GRADE: 2C | Strength of recommendation: weak**

**Quality of evidence: low**

Randomized studies and meta-analyses demonstrate that family members of patients with gastric cancer (GC) exhibit a 2 to 3 times higher risk of developing the disease compared to the general population^118^ ^,^ ^119^. Compared to the general population, they also show a higher incidence of *H. pylori* infection and more severe precancerous histological changes, likely due to two shared risk factors for GC: environmental exposure (*H. pylori* infection) and genetics^120^ ^,^ ^121^. A prospective, randomized, placebo-controlled study involving 1676 family members of GC patients, with a median follow-up of 9.2 years, demonstrated a 55% lower cancer risk in the group receiving antibiotic treatment versus placebo. In this same study, GC risk was 73% lower in the group with confirmed eradication compared to those with persistent *H. pylori* infection^118^. Other observational studies demonstrated that eradication substantially reduced cancer risk compared to persistent infection^120^ ^,^ ^121^. Thus, various international consensuses and guidelines recommend eradicating *H. pylori* in first-degree relatives of gastric cancer patients^5^ ^,^ ^17^ ^,^ ^51^ ^,^ ^122^. A Chinese study further demonstrated that eradication is cost-effective compared to non-eradication^123^. The recommended age to screen for *H. pylori* in family members of GC patients remains unstandardized in international guidelines, but there is a growing consensus that screening should ideally occur before precancerous changes develop^124^ ^,^ ^125^. The recommendation adopted here, with a slight adjustment regarding the minimum age (30 years) to begin endoscopic *H. pylori* screening in family members of GC patients, aligns with the recent guidelines from the European Societies of Gastrointestinal Endoscopy, Helicobacter pylori and Microbiota, and Pathology^87^.

### Statement 12

### Patients undergoing treatment with aspirin (ASA), nonsteroidal anti-inflammatory drugs (NSAIDs), and antiplatelet agents may encounter an elevated risk of developing gastric lesions and gastrointestinal bleeding. In these cases, if possible before initiating treatment, patients should be tested for *H. pylori*, and the infection eradicated.

**Agreement: 100%**

**GRADE: 1A | Strength of recommendation: strong**

**Quality of evidence: high**

In patients with peptic ulcers, the use of NSAIDs and low-dose ASA constitutes a risk factor for gastrointestinal hemorrhage^126^. Furthermore, *H. pylori* infection is also statistically associated with a significant increase in bleeding in these cases. *H. pylori* infection among patients receiving concomitant antithrombotic therapy increases the risk of gastrointestinal bleeding by approximately 1.7 times, and by roughly 8.4 times among patients utilizing antiplatelet agents^51^ ^,^ ^127^. Consequently, treating the *H. pylori* infection is a crucial step in reducing bleeding risk in these cases.

Major gastroenterology consensus guidelines, such as Maastricht VI/Florence, have emphasized that *H. pylori* testing should be prioritized, especially in long-term ASA users^51^. This phenomenon has been analyzed in studies such as the HEAT trial, which included 30,166 patients taking daily doses of ≤325 mg of aspirin and demonstrated a significant reduction in gastrointestinal bleeding with routine *H. pylori* eradication^128^. However, certain study limitations warrant consideration, such as the transient effect of this approach, as the benefit was lost after 2.5 years of follow-up; also, only a small fraction of the studied patients (0.7%) were prescribed dual therapy with antiplatelet and anticoagulant agents. Thus, some controversies persist regarding the ideal population (more or less severe cases) and the most appropriate timing to implement a routine test-and-treat strategy. Regarding patients treated with antiplatelet agents (other than ASA) in the presence of *H. pylori* infection, the increased risks of peptic ulcer bleeding are notable. However, these risks do not manifest when antiplatelet agents are administered in the absence of *H. pylori* ^129^. Anticoagulants do not cause ulcers, but they increase bleeding risks, and the presence of *H. pylori* appears to exacerbate this vulnerability^130^.

### Statement 13

### Testing for and eradicating *H. pylori* are recommended for adults with unexplained iron deficiency anemia and/or immune thrombocytopenic purpura (ITP).

**Agreement: 94%**

**GRADE: 1A | Strength of recommendation: strong**

**Quality of evidence: high**

Iron deficiency is the most common micronutrient deficiency worldwide, affecting nearly 1 in 6 individuals, or approximately 16.7% of the population^131^. Several studies have demonstrated a correlation between chronic *H. pylori* infection and iron deficiency anemia. The mechanisms are diverse and include: a potential interference with iron absorption secondary to hypo- or achlorhydria or reduced gastric ascorbic acid levels; increased hepcidin via inflammation, which reduces iron absorption by enterocytes; bacterial competition for intraluminal iron; and the occurrence of micro-bleeding^132^.

Current clinical guidelines and studies support testing for *H. pylori* infection in patients with unexplained iron deficiency anemia (after investigation with upper GI endoscopy, colonoscopy, and the exclusion of other causes) and recommend treatment if the bacterium is detected^38^ ^,^ ^133^.

A meta-analysis including 15 observational studies^134^ demonstrated that *H. pylori* infection was a risk factor for diminished body iron stores, and infected individuals exhibited a higher risk of developing iron deficiency anemia (odds ratio of 2.8). Another meta-analysis showed that patients with *H. pylori* infections had a 1.72 times higher risk of developing iron deficiency anemia compared to uninfected individuals^135^. Regarding treatment, four interventional meta-analyses suggest that in patients with *H. pylori* infections and iron deficiency anemia, bacterial eradication combined with iron administration is more effective than iron therapy alone. This greater effectiveness translated into higher serum ferritin and hemoglobin levels among patients who received treatment for the infection^136^ ^-^ ^139^. Therefore, the convergence of biological plausibility and clinical evidence of hematological benefit post-eradication supports the treatment recommendation^133^ ^-^ ^139^.

In 1998, Gasbarrini reported a significant increase in platelet counts following *H. pylori* eradication in 8 out of 11 patients with ITP, proposing a pathophysiological link between the infection and ITP^140^. Since then, meta-analyses, prospective series, and observational studies have reported a sustained platelet increase in about 40% to 50% of *H. pylori*-positive patients with ITP undergoing eradication therapy^141^ ^,^ ^142^. A 2018 meta-analysis evaluating 6 clinical trials with 241 patients showed that those who had the infection treated had a higher overall platelet response rate compared to the control group (OR 1.93)^142^. The data show that *H. pylori* eradication facilitates an increase in platelet counts in patients with ITP. However, the benefit is not universal, with variable responses across different geographical regions, which may be tied to strain differences and host-related factors. Brazilian studies in adults and children show benefits in certain patient subgroups^143^ ^,^ ^144^.

International guidelines recommend investigating *H. pylori* in patients with ITP, especially in refractory or recent-onset forms^145^. When the test is positive, bacterial eradication is indicated, given its low cost, good safety profile, and potential hematological response, which may prevent or delay the need for immunosuppression.

Finally, studies investigating the link between vitamin B12 deficiency and *H. pylori* infection, first reported in 1984^146^, have yielded variable results regarding malabsorption^147^ ^-^ ^150^. High-quality studies linking eradication to the resolution of vitamin B12 deficiency anemia are scarce^151^. However, given the potential mechanism (infection-driven atrophy resulting in impaired absorption), its eradication is still recommended by some^51^ ^,^ ^152^, though not all, international consensuses^17^ ^,^ ^38^.

### Statement 14

### *H. pylori* testing and eradication are recommended among patients with GERD on chronic antisecretory therapy (PPIs and P-CABs).

**Agreement: 100%**

**GRADE: 1A | Strength of recommendation: strong**

**Quality of evidence: high**

*H. pylori* eradication does not alter the response to antisecretory drugs^153^, but whether *H. pylori* eradication triggers or worsens pre-existing GERD remains a controversial topic^154^ ^,^ ^155^. Notably, the chronic use of antisecretory agents, such as PPIs, for the maintenance treatment of patients with GERD infected with *H. pylori* facilitates bacterial migration from the antrum to the gastric corpus, leading to corpus-predominant gastritis. Gastric corpus atrophy scores can increase by approximately two to three times when comparing *H. pylori* (+) versus *H. pylori* (-) patients utilizing PPIs^156^. These are two high-risk conditions for gastric cancer development, which can be effectively prevented by early *H. pylori* eradication^157^ ^-^ ^159^.

Further complicating this clinical scenario, hypochlorhydria induced by antisecretory drugs, or parietal cell mass loss induced by gastric atrophy associated with chronic *H. pylori* infection, has also been linked to changes in the gastric microbiome unrelated to *H. pylori*. Among patients with gastric *H. pylori* infection, the interactions between changes in the gastric microbiome, altered gastritis topography with subsequent gastric mucosal atrophy, and precancerous development remain to be fully elucidated^51^ ^,^ ^160^. There appears to be no clinically significant difference in long-term safety between PPIs and P-CABs regarding gastric cancer risk post-eradication^161^.

From a clinical practice standpoint, *H. pylori* eradication improves gastritis among patients who require chronic antisecretory therapy^156^ ^,^ ^162^ ^,^ ^163^.

### Statement 15

### Upper GI endoscopy and an *H. pylori* diagnostic test should precede bariatric surgery, regardless of the type of surgery to be performed, and eradication of the infection is indicated, followed by confirmation of cure.

**Agreement: 100%**

**GRADE: 2C | Strength of recommendation: weak**

**Quality of evidence: very low**

A recent systematic review that included 208 studies totaling 472,511 individuals with obesity found a global estimated *H. pylori* prevalence of 32.3%^164^. Analyzing the 16 Brazilian studies included in this review, covering 2036 individuals, *H. pylori* prevalence was 48.9%, well above the global average. Major international societies for metabolic and bariatric surgery, such as the International Federation for the Surgery of Obesity and Metabolic Disorders (IFSO) and the American Society for Metabolic and Bariatric Surgery (ASMBS), recommend performing preoperative upper GI endoscopy, even among asymptomatic individuals. This approach aims to guide the treatment of modifiable conditions before surgery-such as *H. pylori* infection-alter surgical planning, or even contraindicate the procedure^165^ ^,^ ^166^. In a survey of 96 bariatric surgery experts from 46 countries, two-thirds of the participating clinicians performed endoscopy with biopsy and *H. pylori* eradication, when positive, prior to surgery, and verified eradication^167^. A study involving three bariatric centers in Italy, France, and Lebanon found abnormal preoperative endoscopy findings in 75.6% of patients^168^.

Two recent meta-analyses evaluating the impact of *H. pylori* infection on postoperative complications of sleeve gastrectomy reported conflicting results: one found no statistically significant difference between groups for overall complications (*P*=0.08)^169^, while the other concluded that *H. pylori* infection was correlated with higher overall postoperative complication hazard ratios (*P*=0.007), although noting that definitive causality remains unclear^170^. A study comparing patient-reported postoperative outcomes via questionnaires after sleeve gastrectomy revealed that *H. pylori* infection can negatively impact patient symptoms and experiences, such as more frequent bloating and regurgitation, and exacerbated reflux and postprandial heartburn symptoms, despite medical treatment^171^.

Marginal ulcers represent a persistent concern following gastric bypass. A meta-analysis evaluating predictive factors for this outcome demonstrated that *H. pylori* was a significant predictor for ulcer development (OR=4.97)^172^. In a recent systematic review and meta-analysis assessing the effect of *H. pylori* on bariatric and metabolic surgery complications, the complication prevalence between *H. pylori*-positive/negative patients was not significantly different (*P*>0.05)^173^. However, among patients with *H. pylori* lacking preoperative eradication, the odds ratio (OR) for bleeding was 1.48, for ulcers 6.88, for fistulas 1.73, for strictures 1.13, and for abscesses 3.01. The authors concluded that *H. pylori* infection is associated with potential postoperative complications and therefore requires adequate treatment prior to surgery.

Conversely, an ASMBS publication states that the role of *H. pylori* in marginal ulcer development is not clearly defined, with no strong specific recommendation for or against *H. pylori* testing before metabolic and bariatric surgery to prevent them^174^.

### Statement 16:

### *H. pylori* eradication is recommended if desired by the patient.

**Agreement: 100%**

**GRADE: 2C Strength of recommendation: weak**

**Quality of evidence: very low**

The vast majority of patients who seek medical care following a positive *H. pylori* diagnostic test prefer eradication therapy. The World Gastroenterology Organization Global Guideline on *Helicobacter pylori* states, as a good practice point, that the decision to test for *H. pylori* should only be made with therapeutic intent. The guideline incorporates patient preference, after full consultation with the clinician, as one of the treatment indications^17^. Acceptance necessitates weighing future risks (especially gastric cancer and peptic ulcer disease), acknowledging the possibility of antibiotic adverse events, and committing to performing a test to confirm bacterial eradication. Shared decision-making is recommended for older adults with frailty, when the anticipated clinical benefit is marginal, or when deciding whether to halt or pursue further therapeutic attempts after multiple previous failures, balancing incremental benefit against the inconvenience and re-exposure to antibiotics and acid suppression^175^.

## Working Group 3: diagnosis

## Statement 17:

## The ^13^C-urea breath test (^13^C-UBT) is the gold standard for non-invasive diagnosis and for confirming the eradication of *H. pylori* infection. Monoclonal stool antigen testing is a valid alternative. Serological testing may be indicated in epidemiological studies and specific clinical conditions but has no role in confirming infection eradication. 

**Agreement: 100%**

**GRADE: 1A | Strength of recommendation: strong**

**Quality of evidence: high**

The ^13^C-UBT is the gold standard among non-invasive methods for the diagnosis and confirmation of eradication of *H. pylori* infection, and it has been validated in Brazil for adults and pediatric patients older than 6 years^176^ ^-^ ^178^. It is easy to perform, features variable costs, and exhibits the highest accuracy among non-invasive methods^179^ ^-^ ^181^. The use of citric acid as the test meal is recommended because it delays gastric emptying and prolongs the substrate’s contact with the bacterial urease^51^ ^,^ ^182^. ^13^C-labeled urea developed in Brazil has shown good performance^183^. The ^14^C-urea breath test (^14^C-UBT) has slightly lower accuracy and should be avoided among pediatric and pregnant women^179^ ^-^ ^181^.

Stool antigen testing using ELISA with monoclonal antibodies is a valid alternative to the ^13^C-UBT, with good accuracy in both adults and pediatric patients, and can be used for both diagnosis and eradication confirmation^179^ ^,^ ^180^ ^,^ ^184^ ^,^ ^185^. This assay outperforms tests utilizing polyclonal antibodies and chromatography; the latter may be useful for rapid infection diagnosis-such as pre-recruitment screening followed by confirmatory testing in population-wide treatment programs-but it is not indicated for confirming eradication^185^ ^,^ ^186^. The stool antigen test requires local validation, and its performance can be compromised by inadequate storage or diarrhea^184^ ^,^ ^185^ ^,^ ^187^.

Serological testing is based on detecting anti-*H. pylori* antibodies, primarily IgG and IgA, in blood or urine^188^ ^,^ ^189^. It also requires local validation and can be performed using various techniques; however, the most accurate test is the measurement of IgG in the blood using the ELISA method^188^ ^,^ ^190^. Serology cannot detect active infection, and its main indication is for epidemiological studies. It should not be used to confirm the eradication of *H. pylori* infection^51^ ^,^ ^180^ ^,^ ^188^ ^,^ ^190^. IgG testing via ELISA may be useful as a complementary diagnostic tool in situations involving a reduced bacterial load, such as peptic ulcer bleeding, gastric MALT lymphoma, gastric cancer, gastric atrophy, or recent use of proton pump inhibitors, antibiotics, or bismuth^190^ ^,^ ^191^.

## Statement 18

## Upper GI endoscopy with gastric biopsies for the diagnosis of *H. pylori* infection and mucosal evaluation is indicated among individuals with alarm features; patients aged 40 years and older with dyspeptic symptoms; patients who have experienced failure with antisecretory therapy or the “test-and-treat” strategy; and family members aged 30 years and older of patients with gastric cancer.

**Agreement: 100%**

**GRADE: 2B | Strength of recommendation: weak**

**Quality of evidence: moderate**

The indications and techniques for performing endoscopy with biopsies and *H. pylori* testing are defined in global guidelines. They include patients with dyspepsia, with a minimum age of 45 or 55 years, individuals with alarm features such as weight loss, iron deficiency anemia, gastrointestinal bleeding, a palpable mass, and dysphagia, patients with a family history of gastric cancer in first-degree relatives, and patients who have experienced failure with antisecretory therapy or the “test-and-treat” strategy^51^ ^,^ ^192^. However, although the overall incidence of gastric cancer has been declining, recent studies have shown an upward trend in early-onset gastric cancer before age 40 years in some countries, especially in Latin America (e.g., Mexico, Colombia, Venezuela, and Ecuador)^193^. Considering Brazil’s epidemiological diversity regarding *H. pylori* prevalence and gastric cancer incidence, and recognizing that the timing of *H. pylori* eradication is essential to reduce gastric cancer risk, the indication for upper GI endoscopy in patients with dyspepsia has been suggested at a younger age in certain populations^194^ ^,^ ^195^.

## Statement 19

## Histology is the gold standard for diagnosing *H. pylori* and evaluating the structural integrity of the gastric mucosa. Immunohistochemistry is reserved for special cases. It is recommended to collect at least four biopsies: two from the antrum and two from the gastric corpus, placed in separate, properly labeled specimen containers. Biopsy of the incisura angularis is desirable. The rapid urease test alone can be an alternative diagnostic in resource-limited settings but is not recommended for confirming *H. pylori* eradication. Susceptibility testing (culture or PCR) is reserved for cases of therapeutic resistance.

**Agreement: 100%**

**GRADE: 2B | Strength of recommendation: weak**

**Quality of evidence: moderate**

Upper GI endoscopy combined with biopsy collection is the most reliable diagnostic procedure for evaluating patients with alarm symptoms^51^. Histopathological evaluation offers 95% sensitivity and 99% specificity for diagnosing *H. pylori* infection^51^ ^,^ ^196^. Additionally, it provides complementary information on the gastric mucosa, enabling targeted biopsies of suspicious areas. However, it has limitations, including high costs, inter-observer variability, accuracy affected by the use of proton pump inhibitors or antibiotics, and the need for qualified professionals^197^. Two samples from the antrum, obtained from the lesser and greater curvatures 2 to 3 cm from the pylorus, and two samples from the corpus, from the lesser curvature about 4 cm from the incisura and from the mid-portion of the greater curvature about 8 cm from the cardia, are recommended^54^.

Although the incisura angularis is recognized as the site with the highest incidence of intestinal metaplasia according to the modified Sydney System^54^, sampling this area remains optional. According to the European MAPS III guidelines and the REGAIN study, additional sampling at this anatomical site provides a low diagnostic yield in identifying patients at high risk (OLGA/OLGIM stage III/IV) and does not impact risk classification for intestinal metaplasia or gastric atrophy^50^ ^,^ ^87^. *H. pylori* tends to colonize areas of the gastric mucosa that are still relatively well-preserved. In regions with atrophy, intestinal metaplasia, ulcers, or erosions, bacterial density drops considerably. Consequently, a biopsy taken solely from these altered areas may yield false-negative results^11^ ^,^ ^50^. Samples must be placed in two separate vials, properly labeled with their topographical location. Additional biopsies from lesions or any other visible mucosal abnormalities should be placed in separate, properly labeled vials as well^11^ ^,^ ^50^ ^,^ ^87^ ^,^ ^198^.

The rapid urease test (RUT) is an alternative to histopathology, especially in resource-constrained environments^199^. Two samples (antrum and corpus) are collected, with sensitivity ranging from 80% to 100% and specificity from 97% to 99%^200^. However, sensitivity may decrease among patients undergoing gastrectomy or bariatric surgery, among individuals experiencing active bleeding, and when verifying *H. pylori* eradication therapy (sensitivity drops to around 60%). For this reason, the urease test is not recommended as a stand-alone method for diagnosis in these patient groups^200^ ^,^ ^201^.

Bacterial culture is considered the gold standard for assessing antibiotic susceptibility. To perform this assay, biopsies are collected from the antrum and corpus and must be placed in a sterile container with an appropriate transport medium^201^. The method’s main limitations include the extended duration required to obtain results, low sensitivity for *H. pylori* diagnosis, and limited availability to a few laboratories^51^.

Molecular methods based on nucleic acid amplification via PCR to detect *H. pylori* genetic material from gastric biopsies demonstrate 95% sensitivity and specificity, while also allowing the detection of mutations associated with antibiotic resistance. This type of analysis generally requires additional biopsies but can be performed using samples collected for the rapid urease test^202^. However, the application of these methods in Brazil is still restricted, demanding specialized laboratories, higher costs, and greater technical complexity^5^. It is important to highlight that all invasive methods can produce false-negative results in settings of low bacterial density, recent use of antibiotics, bismuth, or proton pump inhibitors, or in cases of active bleeding^203^ ^,^ ^204^.

Recent studies employing Endofaster-a device that provides real-time biochemical analysis of ammonia concentrations and pH in gastric juice aspirated during endoscopy-have shown good accuracy and, particularly, a high negative predictive value^205^.

## Statement 20

## It is recommended that H2-receptor antagonists be discontinued 2 h prior to diagnostic testing, PPIs and P-CABs 14 days prior, and antibiotics and bismuth compounds 4 weeks prior. Prior discontinuation of the use of sucralfate, alginate, and antacids is not required.

**Agreement: 100%**

**GRADE: 1A | Strength of recommendation: strong**

**Quality of evidence: high**

Various methods are used to detect *H. pylori*, but accuracy can be significantly compromised by medications that reduce bacterial load and/or interfere with urease activity. Antibiotics are the primary cause of false-negative results, as they diminish the active presence of the microorganism. For urea breath tests, recent antibiotic use can cause temporary bacterial suppression or eradication, reducing urease production and compromising test sensitivity. Similarly, in stool antigen testing, decreased excretion of bacterial antigens leads to false-negative results. In invasive methods such as histology, the rapid urease test, and culture, the reduced bacterial density hampers direct detection and can inhibit growth in culture^206^. For this reason, various guidelines and consensuses recommend discontinuing antibiotics for at least 4 weeks before performing these tests in both adults and children/adolescents^5^ ^,^ ^11^ ^,^ ^17^ ^,^ ^38^ ^,^ ^51^ ^,^ ^122^ ^,^ ^207^.

Regarding bismuth salts, their mechanism of action against *H. pylori* is not entirely clear, but they are believed to bind to essential metalloproteins and metalloenzymes, disrupting multiple bacterial pathways^208^. These compounds can exert a bactericidal or bacteriostatic effect, leading to temporary bacterial suppression and reduced urease activity, which facilitates false-negative results in diagnostic tests. A Colombian study estimated that colloidal bismuth subcitrate can cause between 45% to 55% of false-negative results in the ^13^C-UBT and 10% to 15% in stool antigen testing^209^. Thus, it is recommended to avoid bismuth use for at least 4 weeks before performing non-invasive and endoscopic tests, including histology and the rapid urease test^38^.

Proton pump inhibitors (PPIs) and potassium-competitive acid blockers (P-CABs) also exert a direct effect on *H. pylori*. While rarely achieving eradication on their own, they reduce bacterial load and interfere with diagnostic accuracy. In a prospective randomized study, after 14 days of omeprazole 20 mg/daily or esomeprazole 40 mg/day, the sensitivity of the ^13^C-UBT varied from 77.1% to 85.4%, while that of the stool antigen test dropped to 83%, remaining unchanged in the control group on antacids^210^. A Japanese study showed that both vonoprazan and lansoprazole significantly reduced urease activity detected in the ^13^C-UBT, with no changes in the control group^211^. Previous studies and current guidelines recommend stopping PPIs and, by extension, P-CABs for at least 2 weeks prior to breath tests, stool antigen tests, and endoscopic exams, including the urease test, histology, and culture^38^ ^,^ ^212^ ^,^ ^213^.

H2-receptor antagonists do not exhibit significant anti-*H. pylori* activity, and while their precautionary discontinuation a few days before non-invasive tests has been suggested, this practice is not uniform^38^ ^,^ ^51^ ^,^ ^214^ ^-^ ^216^.

Sucralfate can also generate false-negative results by reducing bacterial density and urease activity, as well as by forming a protective barrier over the mucosa that hinders substrate contact with the bacteria. The limited available studies offer conflicting views on its interference with *H. pylori* detection tests. While some studies show up to an 80% reduction in urease activity during its use-reversing upon discontinuation^217^-and potential inhibition of bacterial mucus-degrading enzymes that could compromise histological detection^218^, other studies found no significant difference in ^13^C-UBT results after 14 days of sucralfate alone^219^ or in the urease test following 1.0 g four times daily for 6 weeks in patients with ulcers^220^. Major guidelines make no mention of stopping it prior to *H. pylori* detection tests^5^ ^,^ ^17^ ^,^ ^38^ ^,^ ^51^ ^,^ ^122^. More studies are needed in this area. Alginate works by forming a physical barrier over the gastric contents without altering bacterial density or urease activity. Antacids, due to their low acid-neutralizing capacity and short duration of action, do not significantly affect the sensitivity of breath or stool antigen tests. Neither alginate nor antacids need to be discontinued prior to *H. pylori* diagnostic testing^210^.

## Working Group 4: gastric cancer

## Statement 21

## Serological tests for gastrin-17 and pepsinogens (PGI, PGII, and the PGI/PGII ratio) are non-invasive tools useful for identifying atrophic gastritis and stratifying gastric cancer risk. When combined with anti-*H. pylori* antibodies in populations at high risk for neoplasia, they display good diagnostic accuracy and may be indicated for population screening and clinical monitoring, though they do not replace upper GI endoscopy with biopsy as the gold standard.

**Agreement: 92%**

**GRADE: 2B | Strength of recommendation: weak**

**Quality of evidence: moderate**

Serum levels of pepsinogens (PGI, PGII, and the PGI/PGII ratio) and gastrin play a relevant role in evaluating gastric atrophy, whether secondary to *H. pylori* infection or autoimmunity. They function as non-invasive methods for screening and stratifying populations at risk for gastric cancer. Pepsinogen I is primarily produced by the chief cells located in the mucosa of the gastric corpus and fundus, while pepsinogen II is secreted throughout the stomach and duodenum. In gastric corpus atrophy-a hallmark of autoimmune cases-there is a significant drop in PGI levels and the PGI/PGII ratio, reflecting the loss of oxyntic cells. Values of PGI <70 μg/L and a PGI/PGII ratio <3.0 are highly suggestive of extensive corpus atrophy, exhibiting high sensitivity and specificity, though this can vary depending on the population studied^197^ ^,^ ^221^ ^-^ ^224^.

Gastrin, especially gastrin-17, is primarily produced by antral G cells. In cases of gastric corpus atrophy, the resulting hypochlorhydria triggers a compensatory increase in serum gastrin levels through a feedback mechanism-the classic pattern for patients with autoimmune atrophic gastritis. On the other hand, antral atrophy may present with reduced gastrin-17 levels, which can limit accurate atrophy assessment in cases of atrophic pangastritis, typically seen with *H. pylori* infection. Thus, combining these markers (PGI, PGII, PGI/PGII ratio, and gastrin-17) facilitates the identification of atrophy, suggesting its location (antrum vs. corpus) and inferring its extent and severity^197^ ^,^ ^221^ ^-^ ^223^.

Diagnostic accuracy is highest for moderate to severe corpus atrophy and is limited for mild atrophy or atrophy restricted to the antrum-specifically the patients who exhibit the lowest risk of neoplasia^223^. The presence of *H. pylori* infection can influence the levels of these markers, which must be factored into result interpretation^224^.

By combining the evaluation of these three markers with anti-*H. pylori* antibodies (the so-called “serological panel”), sensitivity can reach up to 75% and specificity up to 95% for diagnosing gastric atrophy. This is useful for stratifying gastric cancer risk and identifying patients who should undergo endoscopy. Its accuracy appears limited in populations with a low risk of neoplasia, and ideally, these markers should be validated locally^223^ ^,^ ^225^ ^-^ ^228^.

Given this evidence, gastrin and pepsinogen tests represent valuable tools in clinical practice. Although they do not replace endoscopy with biopsies from the antrum, corpus, and incisura-the gold standard for diagnosis and staging-they offer a cost-effective alternative for population screening, post-treatment monitoring, and epidemiological studies in regions with high gastric cancer incidence. Furthermore, they are highly useful in settings where endoscopy is unavailable or as an adjunct to endoscopic evaluation^197^ ^,^ ^225^.

## Statement 22

## Patients with high-risk gastric atrophy and/or gastric intestinal metaplasia (GIM) (OLGA/OLGIM III or IV and/or incomplete GIM) are candidates for endoscopic surveillance every 3 years. This interval should be reduced to 1 to 2 years if they have a family history of gastric cancer.

**Agreement: 96%**

**GRADE: 1B | Strength of recommendation: strong**

**Quality of evidence: moderate**

The risk of malignant transformation, especially for intestinal-type gastric carcinoma (GC), progressively increases following the development of gastric atrophy (GA) and gastric intestinal metaplasia (GIM) in the context of chronic gastritis, regardless of GC incidence in the population studied. Meta-analyses and prospective studies with long follow-up periods have demonstrated that these precancerous conditions act as potential monitoring parameters for the secondary prevention of GC^229^ ^,^ ^230^, including the diffuse type^231^.

Chronic gastritis staging systems have been proposed based on the identification of GA and GIM (the OLGA system - Operative Link on Gastritis Assessment) or solely GIM (the OLGIM system - Operative Link on Gastric Intestinal Metaplasia). In both systems, it is mandatory to evaluate at least two biopsy specimens collected from each anatomical site (antrum and corpus), submitted in separate vials. The parameter initially observed is the extent of each process (GA and/or GIM) in each anatomical site separately. It is classified as mild when present in less than 1/3 of the sample, moderate between 1/3 and 2/3, and severe when over 2/3 of the mucosa is involved. For final staging (0 to IV), the extent across both anatomical sites is considered collectively, with more extensive processes classified into categories III and IV^231^ ^,^ ^232^.

Despite the good correlation between staging via both systems, GIM detection shows better intra- and inter-observer agreement compared to GA, favoring the use of OLGIM over OLGA^233^. Accurate histological investigation of GA requires examining the full thickness of the mucosa, from the superficial foveolae down to the muscularis mucosae, necessitating proper specimen orientation during paraffin embedding. GIM evaluation does not require such measures, facilitating verification under the microscope. Regardless, standardized endoscopic evaluation and biopsy collection protocols enhance the likelihood of detecting these conditions in the gastric mucosa^234^.

**FIGURE 1:**
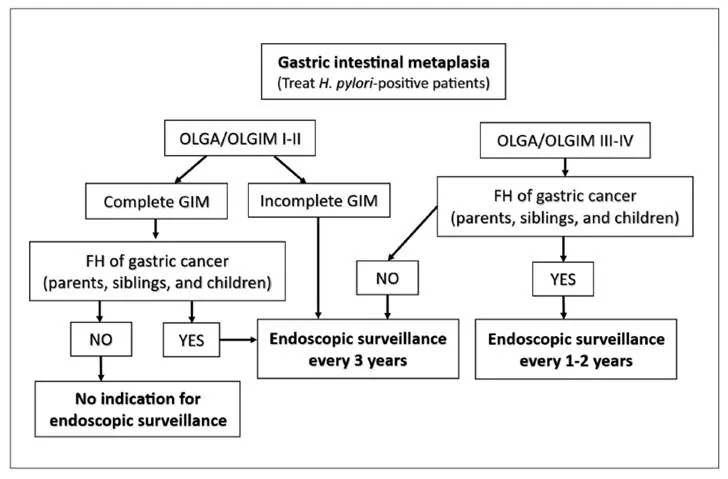
Flowchart for endoscopic surveillance in patients with gastric intestinal metaplasia. OLGA: operative Link on gastritis Assessment. OLGIM: operative link on gastric intestinal metaplasia. FH: family history. GIM: gastric intestinal metaplasia.

Long-term follow-up of patients with GA and GIM demonstrated an elevated probability of progression to dysplasia and malignant transformation in cases classified as OLGA/OLGIM III-IV, justifying endoscopic surveillance in these groups^229^ ^,^ ^230^. In the fourth edition of the Brazilian Consensus on *H. pylori* infection, endoscopies every 2 years were recommended for these patients ^5^. However, based on more recent findings^87^ ^,^ ^90^ ^,^ ^235^, a 3-year interval is now recommended for individuals with high-risk GIM, namely: a) OLGA/OLGIM III-IV; b) extensive GIM; c) incomplete-type GIM. Even if present in a single specimen, the incomplete pattern is considered an independent risk factor for GC in two recent meta-analyses^86^ ^,^ ^236^. If any of these patients has a family member with GC (parents, siblings, or children), it is suggested that the interval be reduced to 1 to 2 years, since family history also represents an independent risk factor. Additionally, if the patient has a family history of GC but their OLGA/OLGIM staging is I-II, endoscopy is suggested every 3 years^237^ ^,^ ^238^. A flowchart summarizing the indications and intervals for endoscopic surveillance in patients with GIM is depicted in Figure 1.

## Statement 23

## The use of an endoscopic classification of atrophy (Kimura-Takemoto) or gastric metaplasia (EGGIM - Endoscopic Grading of Gastric Intestinal Metaplasia) is recommended for the endoscopic staging of precancerous conditions and to stratify the risk of progression to gastric adenocarcinoma. Endoscopic classification must be integrated with histopathological staging. 

**Agreement: 94%**

**GRADE: 2B | Strength of recommendation: weak**

**Quality of evidence: moderate**

Driven by technological advancements and the development of modern endoscopic equipment, upper GI endoscopy plays a fundamental role in visualizing, analyzing, and interpreting the findings that facilitate the visual staging and tissue sampling of the stages comprising the Correa cascade-from the initial infection to the development of intestinal-type gastric adenocarcinoma^239^. The staging of these phases, which include gastritis, atrophy, intestinal metaplasia, dysplasia, and cancer, is achievable through endoscopic examination, aided by increasingly precise visual documentation.

In the absence of *H. pylori* infection, the gastric mucosa presents typical features on white-light endoscopy, namely: a) antrum: flat, smooth mucosa with a light pink hue; b) corpus and fundus: more pronounced folding, especially along the greater curvature, more evident pink coloration, and the presence of a mucus pool; and c) regular arrangement of Collecting Venules (RAC): frequently observed in the gastric corpus. The finding of RAC exhibits a sensitivity of approximately 94% and specificity of 63% for excluding *H. pylori* infection^240^.

*H. pylori* infection can affect both the antrum and the corpus. When it involves both, the progressive risk of developing intestinal-type gastric adenocarcinoma is elevated^239^. When the infection involves the antrum, the mucosa displays erythema, a speckled pattern (spotty redness), and, among young patients, surface nodularity. With gastric corpus involvement, active inflammation can cause thickening and erythema of the folds, making them tortuous, with thick mucus accumulation. In the endoscopic staging of precancerous lesions, it is essential to consider all stages of the Correa cascade previously outlined^241^.

For histological evaluation, the Sydney System, published in 1991 and updated in 1996, standardizes biopsy collection (two from the antrum, one from the incisura angularis, and two from the gastric corpus), correlating etiology, topography, and morphology. This facilitates the assessment of chronic gastritis, location (antrum/corpus), and the presence of atrophy, metaplasia, and *H. pylori* ^54^. To stratify the risk of progression to gastric cancer, the OLGA (Operative Link on Gastritis Assessment) system is used, quantifying the degree and extent of atrophy employing the Sydney System^231^.

The Kimura-Takemoto classification, introduced in 1969^242^, proposes a visual analysis of the gastric mucosa based initially on identifying the “endoscopic atrophic border”-normal mucosa has a pinkish color, whereas atrophic mucosa is pale, with a white/yellowish appearance. It proposes two main types: closed (C) Type - C1: atrophy restricted to the antrum; C2: atrophy extends along the lesser curvature up to the incisura angularis; and C3: extends along the lesser curvature of the corpus without reaching the cardia. Open (O) Type - O1: atrophy reaches the cardia; O2: extends to the anterior wall of the gastric corpus; and O3: reaches the greater curvature. Notably, the extension is parabolic, not linear. The higher the degree of open (O) atrophy, the greater the dysplasia risk. For validation, it is recommended to collect one biopsy from each area designated as C and O.

For the endoscopic evaluation of intestinal metaplasia: using white-light endoscopes with magnification and electronic chromoendoscopy, intestinal metaplasia can be identified as elevated areas (ridges), whitish or grayish in color, displaying a mosaic pattern and mild vascular irregularity. Another technique, in the absence of electronic chromoendoscopy, is the instillation of 1.5% acetic acid, producing an acetowhitening effect on metaplastic areas with high accuracy and specificity^243^.

The EGGIM (Endoscopic Grading of Gastric Intestinal Metaplasia) classification^55^ ^,^ ^244^: uses NBI (Narrow-Band Imaging) to map and score the stomach in three regions (antrum, incisura, and corpus), considering the extent of intestinal metaplasia (≥30% or <30% of the area)^245^.

The OLGIM system (Operative Link on Gastric Intestinal Metaplasia): inspired by OLGA, OLGIM evaluates the extent and distribution of intestinal metaplasia rather than atrophy^232^. Crucially, isolated atrophy or metaplasia in the antrum implies a low risk of neoplasia. However, if it involves the gastric corpus, the risk for intestinal adenocarcinoma progressively increases depending on the extent^246^ ^,^ ^247^.

Dysplasia can be detected visually within areas exhibiting altered coloration or focal irregularities, or randomly, through biopsies in areas of atrophy or metaplasia. When identified, the area should be demarcated using magnification techniques alongside electronic chromoendoscopy^90^ ^,^ ^248^. Chromoendoscopy with 1.5% acetic acid and 1.5% indigo carmine can also be utilized^243^ ^,^ ^245^ ^,^ ^249^.

The most recent guidelines from European Societies (MAPS III, 2025)^87^ recommend that every upper GI endoscopy with chromoendoscopy technology should include screening for early diagnosis of gastric cancer and risk stratification of precancerous conditions. To this end, the use of validated visual classifications such as Kimura-Takemoto (for atrophy) and EGGIM (for metaplasia) is recommended for assessing endoscopic gastric neoplastic risk.

## Statement 24

## In Brazil, population-based *H. pylori* screening for gastric cancer prevention is not recommended. In high-risk groups, screening and treatment of the infection are recommended.

**Agreement: 100%**

**GRADE: 1A | Strength of recommendation: strong**

**Quality of evidence: high**

*H. pylori* eradication is associated with a significant reduction in gastric cancer incidence and mortality^115^. Population-level testing and treatment of *H. pylori* (“screen-and-treat”) has been considered an important strategy for primary prevention. However, several questions remain regarding cost-effectiveness, bacterial resistance, and feasibility^56^ ^,^ ^250^. Over time, randomized clinical trials with long-term follow-up, combined with cost-effectiveness studies conducted in East Asia-an area with high *H. pylori* and gastric cancer prevalence-have strengthened the evidence base, prompting guidelines to become more assertive in recommending this strategy^56^.

Randomized clinical trials in China with up to 25 years of follow-up demonstrated that treating asymptomatic individuals reduced gastric cancer risk by 43% compared to placebo. The benefit was most pronounced among individuals without premalignant changes at the start of the study and with confirmed eradication^251^. In another randomized Chinese trial involving 180,284 individuals aged 25 to 54 years, with up to 11.8 years of follow-up, a significant reduction in gastric cancer incidence was observed in the group that eradicated The bacterium, with no serious adverse events occurring^252^. In the Matsu Islands population program in Taiwan, *H. pylori* prevalence fell from 64% to 15% over 14 years, accompanied by a 53% reduction in incidence and a 25% reduction in gastric cancer mortality compared to historical controls. No relevant changes in antimicrobial resistance rates or increased risks for other digestive tract neoplasms were identified^253^. Projections based on mathematical models show that *H. pylori* eradication therapy is cost-effective for reducing long-term gastric cancer incidence in regions where the neoplasm is highly prevalent^254^.

Population-based eradication is recommended in high-risk regions (annual gastric cancer incidence >20 cases per 100,000) with elevated *H. pylori* prevalence. In this context, a universal approach in adults is indicated, utilizing the urea breath test or stool antigen test, treatment tailored to local resistance profiles, universal confirmation of eradication, endoscopic surveillance for individuals with precancerous lesions, and continuous cost-effectiveness evaluation^38^ ^,^ ^51^ ^,^ ^56^ ^,^ ^195^.

In regions with an intermediate risk of gastric cancer (between 10 and 20 cases per 100,000), there is still no robust evidence to justify a population-level intervention strategy. It is recommended to target *H. pylori* screening and treatment to vulnerable groups, including first-degree relatives of patients with gastric cancer. Clinicians should consider screening and treating adult family members of patients positive for *H. pylori* and integrating national cancer screening programs-such as for colorectal cancer-with gastric cancer and *H. pylori* screening to optimize resources^38^ ^,^ ^56^ ^,^ ^195^ ^,^ ^255^.

In Brazil, the Brazilian National Cancer Institute (INCA) data^256^ for the 2023-2025 triennium show that gastric cancer remains among the cancers with the highest incidence among men, featuring a heterogeneous distribution. Regions such as the North (e.g., State of Pará) and Northeast (e.g., State of Ceará) present high *H. pylori* prevalence and intermediate cancer incidence. This requires individualized strategies, recently systematized for the American continent^257^.

## Statement 25

## Although no approved vaccines exist to prevent the acquisition of *H. pylori* infection or eliminate it in individuals with existing infections, the development of new technologies, such as multi-epitope vaccines, has shown promise in recent preclinical studies.

**Agreement: 96%**

**GRADE: 2C | Strength of recommendation: weak**

**Quality of evidence: low**

Human studies indicate that candidate vaccines against *H. pylori* can induce an immune response and are safe, but they have yet to demonstrate consistent efficacy in preventing or eradicating the infection^258^ ^,^ ^259^. The development of an effective and long-lasting *H. pylori* vaccine remains an unmet need due to the bacterium’s various immune evasion mechanisms and its profound genetic variability, which hinder the creation of a durable protective response in humans through any vaccine^260^ ^-^ ^263^.

The search for new adjuvants in *H. pylori* vaccines has expanded, yet many gaps remain in the successful development of an adjuvanted *H. pylori* vaccine^260^ ^-^ ^261^. Although research and development of *H. pylori* vaccines have achieved some progress over recent decades^264^ ^,^ ^265^, further research is necessary to overcome the immunological and technical challenges identified in clinical studies to date. The development of new vaccine technologies, such as multi-epitope-based vaccines against *H. pylori*, is currently under investigation in experimental trials, providing novel insights for advancing *H. pylori* vaccine development^266^ ^-^ ^268^.

## Working Group 5: treatment And microbiota

## Statement 26

## During the initial patient evaluation, consider: (1) previously used antimicrobial regimens, avoiding the repeated use of clarithromycin and levofloxacin; (2) history of penicillin allergy to clarify questionable cases of true allergy; (3) caution regarding the use of clarithromycin and levofloxacin among patients with QT interval prolongation; (4) choice of antisecretory agent; (5) smoking cessation; and (6) encouragement of treatment adherence. 

**Agreement: 100%**

**GRADE: 1B | Strength of recommendation: strong**

**Quality of evidence: moderate**

The success rate of conventional eradication therapies has declined over the past 2 decades due to rising bacterial resistance, insufficient acid suppression, and poor treatment adherence^269^ ^,^ ^270^. The therapeutic choice must consider allergies, medication availability and cost, and the assessment of local effectiveness data and bacterial resistance, adhering to the principle of avoiding unnecessary drug use. The targeted eradication benchmark is above 90%.

Firstly, a history of prior antimicrobial use, particularly macrolides and quinolones, increases the probability of bacterial resistance. A Chinese population-based study of patients treated with clarithromycin-based triple therapy found an increased risk of retreatment among individuals with a history of prior antibiotic use, especially macrolides, with the risk rising alongside longer antibiotic exposure time^271^. A Korean population study confirmed these findings, revealing significant prior macrolide use over the past decade among patients with clarithromycin-resistant *H. pylori* ^272^. Carothers et al. found that levofloxacin resistance was associated with any prior quinolone use within the past 10 years and increased with a higher number of uses^273^.

Among the antibiotics used in eradication treatment, amoxicillin is one of the most effective, exhibiting low resistance rates^274^. However, approximately 10% of the general population report having a penicillin allergy, yet fewer than 5% of these possess a true allergy^274^ ^,^ ^275^. Patients at moderate and high risk encompass individuals with a history of urticaria, anaphylactic reactions, positive skin tests, recurrent reactions upon use, or hypersensitivity to multiple beta-lactam antibiotics^275^. Patients at low risk include individuals experiencing gastrointestinal symptoms, headaches, or itching without a rash, or those with a history of unknown or doubtful reactions that occurred more than 10 years ago. Skin sensitivity testing and/or referral to an allergist should be indicated for patients considered to be at moderate and high risk^275^.

The use of macrolides or quinolones can be associated with significant QT interval prolongation, a condition linked to potentially fatal ventricular arrhythmias. Consider obtaining an ECG for patients at high risk for QT interval prolongation (e.g., patients with cardiac conditions, individuals with a family history, older adults, patients with electrolyte imbalances, or individuals using antiarrhythmics, antipsychotics, or antidepressants)^274^.

The choice of an antisecretory agent and its proper dosing regimen are essential to optimize treatment efficacy. Effective acid suppression triggers bacterial proliferation, increases antibiotic concentration in the stomach, and prolongs its action on *H. pylori* ^269^ ^,^ ^276^. A systematic review and meta-analysis showed that individuals with a rapid metabolizer phenotype for CYP2C19, the enzyme responsible for 80% to 90% of first-generation PPI metabolism, were associated with an 82% higher probability of therapeutic failure^270^. Jiang et al. suggest that eradication regimens based on P-CABs yielded the optimal eradication rates^276^.

Consistent data show that smoking reduces eradication rates. In a meta-analysis, patients who smoked exhibited an almost twofold higher probability of therapeutic failure than non-smokers^277^. Another meta-analysis confirmed these findings and showed an elevated risk of failure associated with a greater smoking intensity and current use during treatment^278^. Smoking alters CYP2C19 metabolism, increases acidity (which raises the proportion of bacteria in a non-replicative state), and reduces mucosal blood flow, ultimately lowering the efficacy and local availability of antibiotics^278^. In this meta-analysis, no higher risk of failure was observed in studies utilizing vonoprazan. The effect of alcohol consumption remains controversial: a meta-analysis involving 40 studies found no association between alcohol consumption and eradication, except among Asian individuals-a population known to feature altered alcohol metabolism due to aldehyde dehydrogenase deficiency^279^. Alcohol activates gastric acid secretion, reduces amoxicillin absorption, and strongly induces the CYP2C19 enzyme^279^.

Adherence is also a fundamental factor for treatment success. Common barriers include the complexity of eradication regimens, medication intolerance, and poor communication between clinicians and patients^175^. A meta-analysis showed increased eradication rates and treatment adherence when patient education programs were implemented^280^. Two Chinese studies showed significant improvements in adherence and eradication rates utilizing social media and text messages sent to cell phones^281^ ^,^ ^282^.

## Statement 27

## Proton pump inhibitors (PPIs) remain recommended, and the type and dose must be individualized to maximize acid suppression and the consequent therapeutic efficacy. When available, potassium-competitive acid blockers (P-CABs), such as vonoprazan at a dose of 20 mg twice daily, constitute a preferred alternative, especially in treatment regimens containing clarithromycin, amoxicillin, and levofloxacin. 

**Agreement: 100%**

**GRADE: 2B | Strength of recommendation: weak**

**Quality of evidence: moderate**

The use of antisecretory agents, such as proton pump inhibitors (PPIs) and potassium-competitive acid blockers (P-CABs), is fundamental in the treatment of *H. pylori* infection, underpinned by pathophysiological and pharmacokinetic mechanisms. Elevating the intragastric pH (above 4-6) reduces the degradation of acid-sensitive antibiotics such as clarithromycin and amoxicillin, increasing their stability and concentration in the stomach, which enhances their action against *H. pylori*. This bacterium survives at pH 4-8 and multiplies at pH 6-8, a range where it becomes more vulnerable to antibiotics^283^.

PPIs, both first-generation (omeprazole, lansoprazole, dexlansoprazole, and pantoprazole) and second-generation (rabeprazole and esomeprazole), are essential adjuvants in *H. pylori* infection treatment. PPI potency is linked to eradication efficacy, treatment duration, and the choice of antibiotic regimen. It is universally agreed that high doses increase eradication rates, particularly in dual or triple regimens lasting 14 days, especially in populations with a high prevalence of rapid CYP2C19 metabolizers^284^ ^,^ ^285^. The equivalence among different PPIs has been evaluated to define dose potency (Table 2).

**TABLE 2:** Potency of PPIs at different doses, and their omeprazole equivalents (mg).

PPI	Dose (mg)	Omeprazole equivalent (mg)	PPIs Potency*
Pantoprazole	20	4,5	Low dose
Pantoprazole	40	9,0	
Esomeprazole	10	16	
Rabeprazole	10	18	
Omeprazole	20	20	
Lansoprazole	30	27	
Esomeprazole	20	32	Standard dose
Rabeprazole	20	36	
Omeprazole	40	40	
Lansoprazole	60	56	High dose
Omeprazole	60	60	
Esomeprazole	40	64	
Rabeprazole	40	74	

*dose administered twice daily. PPI: proton pump inhibitors. Adapted from Graham DY et al.^285^ and Kirchheiner J et al.^296^.

P-CABs, such as vonoprazan and tegoprazan, among others, have demonstrated faster, more potent, and more prolonged acid suppression compared to PPIs. They are stable in an acidic environment, can be used independently of meals, and do not require conversion to their active form, providing a more immediate effect. With a half-life of 6 to 9 h, in contrast to the 1 to 2 h of PPIs, P-CABs remain available longer, contributing to a more prolonged and effective control of gastric acidity. Furthermore, P-CABs are unaffected by genetic polymorphisms of CYP2C19, being metabolized primarily by CYP3A4/5^161^ ^,^ ^286^.

Direct head-to-head studies have analyzed the efficacy of vonoprazan compared to PPIs in clinical trials. A large trial conducted in the US and Europe compared vonoprazan with lansoprazole in triple therapy (vonoprazan or lansoprazole + amoxicillin + clarithromycin) and dual therapy (vonoprazan + amoxicillin) regimens for 14 days. The results demonstrated that both triple and dual therapies containing vonoprazan were superior to PPI-based triple therapy among patients with clarithromycin-resistant strains (eradication rates of 66% to 70% vs 32%) and among the general population (80.8% and 77.2% vs 68.5%)^287^. Additional randomized studies and meta-analyses, especially from Asia, reinforced the advantage of vonoprazan and tegoprazan in the context of clarithromycin resistance, although they also showed benefits for the general population^288^ ^-^ ^290^.

Currently, no head-to-head studies compare PPIs and P-CABs in combination with levofloxacin and rifabutin for second-line treatments. In bismuth-based quadruple therapy regimens, vonoprazan has demonstrated efficacy similar to PPIs, or with slightly higher eradication rates, and a comparable safety profile^291^ ^-^ ^294^. Vonoprazan has been commercially available since 2020, although it has not been officially approved specifically for anti-*H. pylori* therapy everywhere. In Brazil, its off-label use has been documented in a real-world study developed by the Brazilian Registry on *H. pylori* Management, utilized in 15% of first-line treatments and 26% of retreatments among the 2132 patients analyzed, with promising results^9^ ^,^ ^295^. The recommended doses for vonoprazan and tegoprazan are 20 mg and 50 mg, respectively, administered twice daily^286^ ^,^ ^288^ ^,^ ^293^ ^,^ ^295^.

## Statement 28

## For first-line treatment, the following are indicated: (1) Bismuth quadruple therapy (BQT) with bismuth, metronidazole and tetracycline combined with a PPI or P-CAB (when available) for 10 to 14 days; (2) Triple therapy with clarithromycin and amoxicillin for 14 days, optimized by adding bismuth and/or by substituting the PPI with a P-CAB (when available), twice daily; (3) If bismuth is unavailable, dual therapy with amoxicillin 3 to 4 g/days, divided into three or four doses, combined with a PPI (at a high dose, three or four times daily) or, preferably, a P-CAB (vonoprazan 20 mg, twice daily) when available, for 14 days; (4) Concomitant therapy with a PPI or P-CAB (when available) for 14 days may be an option. 

**Agreement: 100%**

**GRADE: 2C | Strength of recommendation: weak**

**Quality of evidence: low**

Empirical triple therapy (TT) with a PPI has yielded unsatisfactory results^292^ ^,^ ^297^ due to growing bacterial resistance^298^. In the “Brazilian Registry on H. pylori Management”, it was used in 95% of the 1557 first-line treatments, with an eradication rate of 78%^10^. PPI-based TT has been discouraged, except where clarithromycin resistance is <15% or susceptibility is confirmed via prior testing^299^ ^,^ ^300^. Vonoprazan-based TT had higher eradication rates (85% to 89%) than PPI-based TT (71% to 79%) in an Asian meta-analysis and a US/European RCT (66% vs 32%) when treating resistant strains^287^ ^,^ ^292^.

A meta-analysis and two real-world studies de­monstrated that the addition of bismuth to TT (PPI, bismuth-clarithromycin-amoxicillin) optimized first-line outcomes, increasing eradication rates to >90%, comparable to classic bismuth quadruple therapy, even for resistant strains^297^ ^,^ ^299^ ^,^ ^301^.

Two meta-analyses and one real-world study concluded that classic bismuth quadruple therapy (PPI, bismuth, tetracycline, and metronidazole)-administered separately for 14 days or in a single capsule for 10 days consistently yields eradication rates >90% as a first-line treatment, overcoming bacterial resistance^297^ ^,^ ^301^ ^,^ ^302^. Another meta-analysis found no differences in effectiveness when comparing 10 or 14 days of treatment for bismuth quadruple therapies, using either a PPI or a P-CAB as the antisecretory agent^303^. Both bismuth quadruple therapies (classic and amoxicillin-clarithromycin-bismuth) are used in Europe, Asia, and Latin America as preferred first-line options^300^ ^,^ ^301^ ^,^ ^304^, bypassing the need for susceptibility testing even in settings with high bacterial resistance^302^. Aside from frequently reported adverse effects^301^, bismuth salts are unavailable in many countries^298^ and, in Brazil, can still only be obtained exclusively through compounding pharmacies.

Dual therapy (DT) pairs high-dose amoxicillin with a PPI (high dose, three or four times daily)-known as high-dose dual therapy (HDDT)^305^-or vonoprazan (20 mg twice daily)^306^. The amoxicillin dosing is 750 to 1000 mg three or four times daily for 10 to 14 days^306^ ^,^ ^307^. Particularly for patients with obesity, 1 g of amoxicillin four times daily should be used^308^.

HDDT has been employed with favorable results across various regions worldwide, being studied most intensively in Asia. Two recent meta-analyses-one evaluating 14 randomized controlled studies involving 5121 patients-both showed intention-to-treat (ITT) eradication rates of 86% to 87%^305^ ^,^ ^307^. In Latin America, a Colombian randomized study^309^ achieved an 89% eradication rate by ITT, and a Chilean observational study involving 126 patients achieved a 92% eradication rate by ITT^310^. In Europe, a Portuguese observational study involving 100 patients treated with HDDT as a first-line or retreatment regimen observed a 96% eradication rate by ITT analysis^311^.

Vonoprazan-based DT has also shown highly favorable performance as a first-line option in Asia. Two recent meta-analyses, mostly involving randomized controlled studies, found ITT eradication rates between 86% and 89%^306^ ^,^ ^312^. The efficacy of HDDT^305^ and vonoprazan-based DT^306^ ^,^ ^312^ was comparable to that of bismuth quadruple therapies. Both HDDT^313^ and vonoprazan-based DT^287^ ^,^ ^293^ are largely unaffected by bacterial resistance. Advantages of DT include the use of only two drugs, a low rate of adverse events, and a lower cost compared to the other regimens considered^305^ ^-^ ^307^ ^,^ ^312^. Studies remain scarce on vonoprazan-based DT in the West. A US/European randomized controlled study showed 77.2% eradication with vonoprazan-based DT for 14 days^287^ and, interestingly, two large recent Taiwanese randomized studies showed eradication rates between 83% and 84% with this regimen^288^ ^,^ ^314^. A potential limitation regarding the widespread recommendation for DT is the report of emerging amoxicillin resistance >70% in some African countries and >15% in some Asian countries (Vietnam and Iran)^298^.

Concomitant therapy with clarithromycin, amoxicillin, and metronidazole for 14 days is an option where bismuth and susceptibility testing are unavailable, and where clarithromycin resistance is high^300^. This approach demonstrated eradication rates >90% in two large real-world registries^315^ ^,^ ^316^, with good performance against resistant strains^299^. However, dual resistance to clarithromycin and metronidazole can compromise effectiveness^299^. Concomitant therapy utilizes one or two potentially ineffective drugs, which may contribute to increased local resistance^300^.

Sequential and hybrid/reverse therapies also warrant consideration^297^, alongside a new kit containing omeprazole, rifabutin, and amoxicillin, approved for first-line treatment in the US^317^ (Table 3).

**TABLE 3:** Recommended schemes as first-line in patients with *H. pylori* infection.

Regimen	Drugs	Dosing frequency	Duration (days)	Quality of evidence
**If bismuth is available**				
**BTM+PPI/P-CAB**	Bismuth subcitrate 240mg	b.i.d	10^#^-14	Strong (moderate quality of evidence)
	Tetracycline 500mg^a^	q.i.d		
	Metronidazole 400-500mg	q.i.d		
	PPI^b^(standard dose)^c^ or P-CAB	b.i.d		
**BCA+PPI/P-CAB**	Bismuth subcitrate 240mg	b.i.d	14	Weak (low quality of evidence)
	Clarithromycin 500mg	b.i.d		
	Amoxicillin 1000mg	b.i.d		
	PPI (standard dose) or P-CAB	b.i.d		
**If bismuth is unavailable**				
**CA+ P-CAB**	Clarithromycin 500mg	b.i.d	14	Weak (low quality of evidence)
	Amoxicillin 1000mg	b.i.d		
	P-CAB	b.i.d		
**A+PPI/P-CAB**	Amoxicillin 750-1000mg	t.i.d or q.i.d	14	Weak (low quality of evidence)
	IBP (high dose)^d^ or	t.i.d or q.i.d		
	P-CAB	b.i.d		
**CAM + PPI/P-CAB**	Clarithromycin 500mg	b.i.d	14	Weak (low quality of evidence)
	Amoxicillin 1000mg	b.i.d		
	Metronidazole 500mg	q.i.d		
	IBP (standard dose) or P-CAB	b.i.d		

B: bismuth subcitrate. T: tetracycline. M: metronidazole. C: clarithromycin. A: amoxicillin. ^#^Single-capsule of bismuth subcitrate (140mg), metronidazole (125mg), and tetracycline (125mg) is only dispensed as a 10-day regimen kit (total dosage: 03 capsules 4 times daily plus PPI for 10 days); P-CAB, potassium-competitive acid blocker (i.e, vonoprazan 20mg b.i.d); PPI, proton pump inhibitor; b.i.d., twice daily; q.i.d., 4 times daily; t.i.d., 3 times daily. ^a^Doxycycline is not a recommended substitution for tetracycline. ^b^PPI should be dosed 30-60 minutes before a meal. ^c^Standard dose PPI: 32-40 mg omeprazole equivalents, two times per day (i.e., 40 mg omeprazole equivalents, two times per day). ^d^High dose PPI: 54-128 mg omeprazole equivalents, two times per day (i.e., 60 mg omeprazole equivalents, two times per day).

## Statement 29

## For second-line treatment, it is recommended not to repeat the same regimen used in the first line. The following are indicated: (1) bismuth quadruple therapy with metronidazole and tetracycline combined with a PPI or P-CAB (when available) for 10 to 14 days; (2) amoxicillin-levofloxacin-bismuth regimen combined with a PPI or P-CAB (when available) for 14 days; (3) if bismuth is unavailable, dual therapy with amoxicillin 3 to 4 g/day divided into three or four doses combined with a PPI (at a high dose, three or four times daily) or, preferably, a P-CAB (vonoprazan 20 mg, twice daily) when available, for 14 days. 

**Agreement: 100%**

**GRADE: 2C | Strength of recommendation: weak**

**Quality of evidence: low**

Following the failure of clarithromycin-based triple therapy, the most recommended therapeutic option is levofloxacin-amoxicillin-PPI (LA-PPI) therapy for 10 to 14 days^51^. A European systematic review on retreatment found the 14 days therapy superior to the 10 days therapy (87% vs 72%)^318^. To overcome rising levofloxacin resistance, adding bismuth to the regimen has been proposed^319^. A systematic review with network meta-analysis evaluating different second-line therapies showed that levofloxacin-bismuth quadruple therapy for at least 10 days yielded optimal results, backed by moderate-level evidence, when compared to various 7 days triple therapies, with or without quinolones^320^. Another systematic review and meta-analysis included 16 second-line studies post-failure of non-bismuth quadruple regimens (sequential or concomitant) and evaluated the outcomes of different quinolone therapies^321^. In this review, only two studies evaluated the levofloxacin-bismuth quadruple regimen: one for 14 days using LA-PPI-bismuth (93% efficacy) and another using levofloxacin-tetracycline-PPI-bismuth for 10 days, achieving a 96% cure rate, though both featured very small sample sizes (69 and 24 patients, respectively)^321^. Data from the European Registry-though not direct comparisons-involving over 5000 patients on second-line therapies showed that the LA-PPI-bismuth regimen reached 88% effectiveness, whereas LA-PPI therapy reached 81%^322^. In Brazil, results from the Hp-BraReg analyzing 386 second-line retreatments showed that the LA-PPI regimen for 10 or 14 days had suboptimal results (57% and 73%, respectively)^9^. Conversely, adding bismuth to the 14 days LA-PPI regimen optimized results, achieving a 100% cure rate (95%CI 88-100%)^323^. High doses (>500 mg) and low doses (≤500 mg) of levofloxacin per day tend to show similar results^323^.

For retreatment, empirical BQT is recommended, particularly in areas with high quinolone resistance^38^ ^,^ ^51^. The growing rate of levofloxacin resistance and warnings from regulatory agencies led the North American consensus to recommend BQT as the preferred second-line therapy, reserving levofloxacin for susceptibility-guided therapy^38^ ^,^ ^324^. BQT can be prescribed with the drugs individually or in a single capsule (“three-in-one”) containing bismuth, metronidazole, and tetracycline^325^. An Asian meta-analysis of retreatment studies showed that BQT for 10 to 14 days had an eradication rate of 82% vs 76% for 7 days^326^. The ideal duration of this regimen has yet to be fully defined. Few studies have compared BQT regimens of 14 and 10 days. A recent meta-analysis demonstrated that the eradication rate of the 10 days BQT regimen was statistically similar to the 14 days one (RR 0.98; 95%CI 0.95-1.00)^303^. A single-center, non-randomized US-based first-line study found 14 days BQT eradication rates of 87%, whereas 10 days BQT reached 77%^327^.

BQT regimens can cause adverse effects in up to 40% of cases, generally mild and short-lived^38^. Adverse effects and treatment adherence are comparable between 10 and 14 days therapies^303^.

A European systematic review found a 75.6% success rate for BQT in the second line^318^. In that study, the single-capsule BQT regimen for 10 days, currently unavailable in Brazil, achieved higher rates (89%, 95%CI 87.4-91.0% by intention-to-treat) than those obtained by traditional BQT (76%, 95%CI 66.1-85.1%)^318^.

Studies on BQT indicate a trend towards better efficacy with tetracycline compared to doxycycline. In the first line, eradication was 67% to 70% with doxycycline and 85% with tetracycline^328^. In the Hp-EuReg, third-line rates were 65% versus 76%, trending statistically in favor of tetracycline^328^.

When the regimens suggested above are unavailable, dual therapy with high-dose amoxicillin can be considered an option, although few studies provide confirmatory results in the Western world. In a Portuguese study involving patients undergoing retreatment, dual therapy with high-dose amoxicillin (3 g/ day divided into four doses) and a PPI (esomeprazole 40 mg twice daily) for 14 days outperformed the 10 days BQT regimen (100% and 62.5%, respectively; *P*=.028 per protocol and by intention-to-treat)^311^. In the Brazilian Registry, out of 386 patients undergoing retreatment evaluated, 24 received dual therapy with amoxicillin (≥3 g/day in three or more doses) and vonoprazan 20 mg twice daily, with a 75% cure rate^9^. These results must be further evaluated in Western studies with larger sample sizes (Table 4).

**TABLE 4:** Recommended schemes as second-line in patients with *H. pylori* infection.

Regimen	Drugs	Dosing frequency	Duration (days)	Quality of evidence
**BTM+IBP/ P-CAB**	Bismuth subcitrate 240mg	b.i.d	10^#^-14	Strong (moderate quality of evidence)
	Tetracycline 500mg^a^	q.i.d		
	Metronidazole 400-500mg	q.i.d		
	PPI^b^(standard dose)^c^ or P-CAB	b.i.d		
**BLA+IBP/ P-CAB**	Bismuth subcitrate 240mg	b.i.d	14	Weak (low quality of evidence)
	Levofloxacin 500mg	m.i.d		
	Amoxicillin 1000mg	b.i.d		
	PPI (standard dose) or P-CAB	b.i.d		
**A+IBP/P-CAB**	Amoxicillin 750-1000mg	t.i.d or q.i.d	14	Weak (low quality of evidence)
	IBP (high dose) ^d^ or	t.i.d or q.i.d		
	P-CAB	b.i.d		

B: bismuth subcitrate. T: tetracycline. M: metronidazole. C: clarithromycin. A: amoxicillin. L: levofloxacin. ^#^Single-capsule of bismuth subnitrato (140mg), metronidazole (125mg), and tetracycline (125mg) is only dispensed as a 10-day regimen kit (total dosage: 03 capsules 4 times daily plus PPI for 10 days); P-CAB, potassium-competitive acid blocker (i.e, vonoprazan 20mg b.i.d); PPI, proton pump inhibitor; m.i.d, once daily; b.i.d, twice daily; q.i.d, 4 times daily; t.i.d, 3 times daily. ^a^Doxycycline is not a recommended substitution for tetracycline. ^b^PPI should be dosed 30-60 minutes before a meal. ^c^Standard dose PPI: 32-40 mg omeprazole equivalents, two times per day (i.e., 40 mg omeprazole equivalents, two times per day). ^d^High dose PPI: 54-128 mg omeprazole equivalents, two times per day (i.e., 60 mg omeprazole equivalents, two times per day).

## Statement 30

## For rescue regimens, it is recommended not to repeat previously used regimens containing clarithromycin or levofloxacin. The following are indicated: (1) bismuth quadruple therapy composed of bismuth, tetracycline, and metronidazole combined with a PPI or P-CAB (when available) for 10 to 14 days; (2) triple therapy with rifabutin and amoxicillin combined with a PPI or P-CAB (when available) for 14 days, especially when bismuth quadruple therapy is unavailable or has already been used; (3) in the absence of bismuth and rifabutin, dual therapy with amoxicillin 3 to 4 g/day divided into three or four doses combined with a PPI (at a high dose, three or four times daily) or, preferably, a *P*-CAB (vonoprazan 20 mg, twice daily) when available, for 14 days, though Western confirmatory studies are still lacking.

**Agreement: 100%**

**GRADE: 2C | Strength of recommendation: weak**

**Quality of evidence: low**

Following the failure of first- and second-line treatments to eradicate *H. pylori*, the choice of rescue regimen must account for previously used antibiotic regimens, allergies, local drug availability, costs, and, if possible, local and individual resistance profiles. The main options include:

1 - Bismuth quadruple therapy (see recommendations 28 and 29): this is the preferred option for patients with a history of prior treatment, particularly when the resistance profile is unknown or multiple past failures have occurred. Due to the low rates of bacterial resistance to these antibiotics in the regimen, it can even be repeated as a rescue treatment by utilizing a high dose of metronidazole. The lack of commercially available bismuth in standard retail pharmacies (though available via compounding pharmacies) remains a problem affecting regions where approximately one billion people live^298^.

2 - Rifabutin triple therapy: comprising a PPI, amoxicillin, and rifabutin for 10 to 14 days, this is an alternative when bismuth quadruple therapy is unavailable, was previously used, or in cases of tetracycline allergy. In a recent randomized study of patients who underwent two prior therapeutic regimens, rifabutin triple therapy demonstrated eradication rates of 89.0% by intention-to-treat (ITT) and 94.0% per protocol (PP)-results non-inferior to BQT, even in populations with high resistance to clarithromycin, levofloxacin, and metronidazole. Adherence was superior, and total and moderate/severe adverse events were significantly lower in the rifabutin group (26.4% and 14.3%, respectively) compared to BQT (54.4% and 28.6%)^329^. Three systematic reviews reinforce the efficacy of the rifabutin, amoxicillin, and PPI combination in rescue therapies, demonstrating average ITT eradication rates of 71% to 80%^330^ ^-^ ^332^. Initial studies with small sample sizes suggest that combining vonoprazan with rifabutin and amoxicillin is effective and safe, despite methodological limitations^9^ ^,^ ^333^ ^,^ ^334^. A pilot study showed that adding bismuth subcitrate to the rifabutin triple regimen significantly increased the eradication rate without relevant adverse effects^335^. The most heavily studied regimens consist of rifabutin 150 mg twice daily, amoxicillin 1 g twice daily, and a PPI, for 10 to 14 days, with 14 days being the recommended duration^329^ ^-^ ^331^. Rifabutin resistance remains rare, typically under 1%^335^ ^-^ ^337^. The regimen is well-tolerated, with fever, nausea, vomiting, and reversible bone marrow suppression being the most relevant side effects. A complete blood count is recommended during treatment^331^ ^,^ ^337^. Within the Brazilian context, rifabutin is not available on the Brazilian market, necessitating importation. However, it is listed on the Brazilian Unified Health System (SUS) National List of Essential Medicines (RENAME), though its use is strategically restricted to treating resistant mycobacterial infections^338^.

3 - Dual therapy with a PPI or vonoprazan and amoxicillin: recent Asian studies have shown variable results utilizing the combination of high-dose amoxicillin and a PPI as a rescue therapy. Randomized studies and Asian systematic reviews show ITT eradication rates between 73% and 94%^307^ ^,^ ^339^ ^,^ ^340^, with a low rate of adverse effects, even among patients who underwent multiple previous treatment failures. However, Western studies on this rescue regimen remain limited. In a German randomized non-inferiority trial involving patients with prior treatment failure and persistent infection by clarithromycin-resistant and metronidazole-susceptible strains, dual therapy using omeprazole 40 mg and amoxicillin 750 mg, both four times daily, showed eradication rates of 75.6%, non-inferior to bismuth quadruple therapy^341^. A real-world study coordinated by the European Registry on *H. pylori* Management analyzed 41 patients undergoing rescue treatment (2nd to 6th line) with amoxicillin 3.0 g/day and PPIs in varying formulations and doses for 14 days, achieving an ITT effectiveness of only 51%^342^.

While dual therapy with a PPI and amoxicillin can be considered a simplified alternative for treating *H. pylori* in rescue contexts, the most robust available evidence refers to the use of vonoprazan alongside high-dose amoxicillin. In a Chinese real-world retrospective study involving 186 patients with up to seven prior failures, the eradication rate using vonoprazan (20 to 40 mg/day) combined with amoxicillin (3.0 g/day) for 14 days was 92.5% (95%CI: 87.4-95.7%) with mild, self-limiting adverse effects (7.5%), regardless of the number of previous treatments or the regimens previously utilized^343^. A randomized multicenter study with patients who underwent multiple eradication attempts showed that dual therapy with high-dose vonoprazan and amoxicillin for 14 days was non-inferior to BQT, achieving an ITT eradication rate of 73.8% alongside good tolerability^344^. A recent meta-analysis confirms these findings in Asian populations, showing eradication rates above 90% even in rescue scenarios, paired with a favorable safety profile^306^. To date, robust Western studies specifically evaluating dual therapy with vonoprazan and amoxicillin in rescue treatments are absent. In Brazil, interim real-world data from the Hp-BraReg showed that 36 out of 186 patients who underwent three or more *H. pylori* eradication attempts were treated with vonoprazan and high-dose amoxicillin, yielding a modified ITT of 100%, alongside good tolerability and mild adverse effects^9^ ^,^ ^295^ (Table 5).

**Table:** 

Regimen	Drugs	Dosing frequency	Duration (days)	Quality of evidence
**If bismuth is available**				
**BTM+IBP/P-CAB**	Bismuth subcitrate 240mg	b.i.d	10^#^-14	Strong (moderate quality of evidence)
	Tetracycline 500mg^a^	q.i.d		
	Metronidazole 400-500mg	q.i.d		
	PPI^b^(standard dose)^c^ or PCAB	b.i.d		
**If bismuth is unavailable**				
**RA+IBP/P-CAB**	Rifabutin 150mg	b.i.d	14	Weak (low quality of evidence)
	Amoxicillin 1000mg	b.i.d		
	PPI (standard dose) or PCAB	b.i.d		
**A+IBP/P-CAB**	Amoxicillin 750-1000mg	t.i.d or q.i.d	14	Weak (low quality of evidence)
	IBP (high dose)^d^ or	t.i.d or q.i.d		
	PCAB	b.i.d		

B: bismuth subcitrate. T: tetracycline. M: metronidazole. C: clarithromycin. A: amoxicillin. R: rifabutin. ^#^Single-capsule of bismuth subnitrato (140mg), metronidazole (125mg), and tetracycline (125mg) is only dispensed as a 10-day regimen kit (total dosage: 03 capsules 4 times daily plus PPI for 10 days). PCAB, potassium-competitive acid blocker (i.e, vonoprazan 20mg b.i.d). PPI, proton pump inhibitor; b.i.d, twice daily; q.i.d, 4 times daily; t.i.d, 3 times daily. ^a^Doxycycline is not a recommended substitution for tetracycline. ^b^PPI should be dosed 30-60 minutes before a meal. ^c^Standard dose PPI: 32-40 mg omeprazole equivalents, two times per day (i.e., 40 mg omeprazole equivalents, two times per day). ^d^High dose PPI: 54-128 mg omeprazole equivalents, two times per day (i.e., 60 mg omeprazole equivalents, two times per day).

## Statement 31

## Triple therapy with PPI-amoxicillin-clarithromycin and PPI-amoxicillin-levofloxacin should ideally be employed following susceptibility testing, where available. After three therapeutic failures, susceptibility testing should be used when available.

**Agreement: 100%**

**GRADE: 2B | Strength of recommendation: weak**

**Quality of evidence: moderate**

The increase in *H. pylori* resistance rates demands new treatment strategies, including methods to evaluate antibiotic susceptibility^298^. However, controversies remain regarding the superiority of susceptibility-guided therapy over empirical therapy and whether it is cost-effective^302^ ^,^ ^345^.

Among phenotypic techniques, the agar dilution method is considered the gold standard. However, it is labor-intensive, time-consuming, and ill-suited for infections caused by a small number of strains, complicating routine use^346^. Alternative methods include disk diffusion and the E-test, the latter of which is increasingly recommended^347^ ^,^ ^348^.

Yet, phenotypic tests still face controversies. The European Committee on Antimicrobial Susceptibility Testing validated the E-test and standardized the minimum inhibitory concentration values for six antimicrobials (amoxicillin, clarithromycin, levofloxacin, metronidazole, rifampicin, and tetracycline)^349^. In the United States, the Clinical and Laboratory Standards Institute (CLSI) solely recommends the agar dilution test and has only established resistance criteria for clarithromycin^350^. In Brazil, no governmental body has established resistance criteria for phenotypic tests. Beyond the methodological hurdles, phenotypic tests suffer from limited availability, even in developed countries^298^.

As alternatives to culture, genotypic tests employ molecular methods to identify point mutations in *H. pylori* associated with resistance (polymerase chain reaction-PCR or next-generation sequencing)^351^. These methods are faster and can utilize gastric biopsies, gastric aspirates, or stool samples, although stool sampling remains unvalidated^298^. Fecal genotypic testing is under development and could be employed as a non-invasive molecular method. A meta-analysis of 26 studies found that despite a specificity of 96%, sensitivity was only 71%^352^. The primary limitation of the stool method appears to be the presence of PCR inhibitors, leading to an increase in false-negative results. Genotypic methods are not yet commercially available in many countries, including Brazil^298^.

Phenotypic and genotypic methods have been compared and show good correlation, particularly regarding clarithromycin and levofloxacin resistance-the drugs that most impact therapeutic failure^352^ ^,^ ^353^.

Clarithromycin resistance is typically driven by point mutations in the 23S rRNA gene, the most important being A2143G, A2142G, and A2142C^354^. In a meta-analysis comparing guided versus empirical therapy, results for clarithromycin triple therapy favored guided therapy (87% vs 78%), especially in areas with clarithromycin resistance >20%^302^.

Mutations in the gyrA and gyrB genes confer levofloxacin resistance, detectable via genotypic methods^355^. In a meta-analysis, levofloxacin-based triple regimens showed 81% efficacy against susceptible strains and 36% against mutants^323^. Rifabutin resistance involves the rpoB gene; amoxicillin resistance stems from mutations in PBP1A; and tetracycline resistance involves 16S rRNA. Resistance levels for rifabutin, amoxicillin, and tetracycline remain low and stable^355^. Metronidazole resistance usually stems from rdxA mutations, but doses >1.5 g/day within a 14 days bismuth quadruple therapy are capable of overcoming it^356^. In a meta-analysis, bismuth quadruple therapy showed similar efficacy whether empirical or guided, and it was minimally affected by prior antibiotic use, largely due to the rarity of tetracycline and amoxicillin resistance and the limited impact of metronidazole resistance^357^.

Among patients with a history of prior exposure to various antibiotics, particularly macrolides and quinolones for other infections, susceptibility testing may be considered, given the well-established risk of higher resistance^358^.

Beyond bacterial resistance, other factors implicated in therapeutic failure include treatment adherence, inadequate acid suppression, smoking, the expression of efflux pumps, and biofilm formation^175^. Strategies to overcome biofilm formation have been attempted, such as using N-acetylcysteine (NAC)^359^, though a meta-analysis showed no superiority when NAC was added to the standard therapeutic regimen^360^. In practice, overcoming the biofilm is optimally achieved by employing bismuth, maximizing optimized acid suppression, ensuring treatment adherence, and avoiding the repetition of antimicrobial regimens prone to higher resistance, such as clarithromycin and quinolones^361^.

## Statement 32

## For patients with a penicillin allergy, the recommended first-line treatments are: a) Bismuth quadruple therapy with metronidazole and tetracycline combined with a PPI or P-CAB (when available) for 10 to 14 days; b) Dual therapy with tetracycline and a P-CAB (when available) for 14 days. For second-line treatment, one of the previously unused options can be employed, or alternatively, a 14 days triple therapy using a PPI, quinolone, and clarithromycin. 

**Agreement: 100%**

**GRADE: 2B | Strength of recommendation: weak**

**Quality of evidence: moderate**

Amoxicillin is a component of the most effective antimicrobial regimens for *H. pylori* eradication. As such, penicillin allergy is a major clinical challenge.

The global prevalence of penicillin allergy is 9.4% (95% CI 8.4% to 10.4%), with Latin American countries underrepresented in the research^362^. Confirmatory tests for true immune-mediated hypersensitivity-such as skin tests and specific IgE testing-are rarely performed and offer limited diagnostic accuracy^363^ ^,^ ^364^. For patients with a history of penicillin allergy, treatment options must exclude amoxicillin and other penicillins.

Another limitation is the rapid global rise in resistance to clarithromycin, fluoroquinolones, and nitroimidazoles^298^, already demonstrated across the five geographic regions of Brazil^7^.

For first-line treatment among patients with a penicillin allergy, classic 14 days bismuth quadruple therapy is the most consistent recommendation, demonstrating eradication rates around 89% to 90%^51^ ^,^ ^365^ ^-^ ^367^ and is considered adequate in regions with high clarithromycin and metronidazole resistance^51^. Another novel option investigated the comparison of the rapid and potent acid inhibition of vonoprazan versus rabeprazole within a modified quadruple therapy containing bismuth, metronidazole, and doxycycline. The outcome was non-inferiority, with ITT eradication rates of 90.4% versus 71.1%, respectively^293^. Just as dual therapy with amoxicillin and a P-CAB has been used for patients without an amoxicillin allergy history, the first randomized clinical trial in 300 patients with a penicillin allergy utilized vonoprazan and tetracycline (VT) for 14 days. The ITT eradication rate for VT versus classic BQT was 92% vs 89.3%, respectively, demonstrating non-inferiority alongside fewer adverse events^368^.

For second-line treatment among patients with a penicillin allergy, after reviewing the initial antibiotic regimen used, the options are classic bismuth quadruple therapy and triple therapy containing a PPI, clarithromycin, and a quinolone (e.g., levofloxacin), demonstrating eradication rates ranging from 64% in a multicenter prospective study^369^ to 80% to 100% in reviews of retrospective studies^365^ ^,^ ^366^.

Following a second failure, in rescue treatments, triple therapy with clarithromycin and metronidazole is indicated, as is the possibility of repeating classic triple therapy using high-dose metronidazole (1.5 to 1.6 g/day) for 14 days^370^.

Amoxicillin-free empirical treatments have used various antibiotic regimens, especially in regions with high antibiotic resistance and limited medication availability, relying on agents such as cefuroxime, rifabutin, and furazolidone^365^ ^,^ ^366^ ^,^ ^371^ ^,^ ^372^.

Among cases of refractoriness, whenever possible, the penicillin allergy should be confirmed through testing (skin tests and specific IgE)^362^ ^,^ ^363^. Clinicians must ascertain which antibiotics were previously used and, whenever possible, perform antimicrobial susceptibility testing or cultures to guide antibiotic selection^366^ (Table 6).

**TABLE 6:** Recommended treatment regimens for patients with penicillin allergy.

Regimen	Drugs	Dosing frequency	Duration (days)	Quality of evidence
**If bismuth is available**				
**BTM+PPI/P-CAB**	Bismuth subcitrate 240mg	b.i.d	^#^10-14	Strong (moderate quality of evidence)
	Tetracycline 500mg^a^	q.i.d		
	Metronidazole 400-500mg	q.i.d		
	PPI^b^(standard dose)^c^ or P-CAB	b.i.d		
**If bismuth is unavailable**				
**T+P-CAB**	Tetracycline 500mg	t.i.d or q.i.d	14	Conditional (low quality of evidence)
	P-CAB	b.i.d		
**CL+PPI/P-CAB**	Clarithromycin	b.i.d	14	Conditional (low quality of evidence)
	Levofloxacin	m.i.d		
	PPI^b^(standard dose)^c^ or P-CAB	b.i.d		

B: bismuth subcitrate. T: tetracycline. M: metronidazole. C: clarithromycin. L: levofloxacin. ^#^Single-capsule of bismuth subcitrate (140mg), metronidazole (125mg), and tetracycline (125mg) is only dispensed as a 10-day regimen kit (total dosage: 03 capsules 4 times daily plus PPI for 10 days). P-CAB, potassium-competitive acid blocker (i.e, vonoprazan 20mg b.i.d). PPI, proton pump inhibitor; m.i.d, once daily; b.i.d, twice daily; q.i.d, 4 times daily; t.i.d, 3 times daily. ^a^Doxycycline is not a recommended substitution for tetracycline. ^b^PPI should be dosed 30-60 minutes before a meal. ^c^Standard dose PPI: 32-40 mg omeprazole equivalents, two times per day (i.e., 40 mg omeprazole equivalents, two times per day).

## Statement 33

## Specific probiotic strains may exert a beneficial effect during anti-*H. pylori* treatment by increasing eradication rates and reducing side effects caused by antibiotics. However, further studies are needed to better define the specific probiotic strains, required dosage, ideal timing, and duration of supplementation.

**Agreement: 91%**

**GRADE: 2B | Strength of recommendation: weak**

**Quality of evidence: moderate**

Various therapeutic regimens, relying on the combination of antibiotics and antisecretory drugs, are conventionally recommended for *H. pylori* treatment; however, in recent years, a significant reduction in bacterial eradication rates has been observed^5^ ^,^ ^51^. Consequently, recent research has explored alternative options, and several options have shown significant promise, such as adding probiotics to traditional anti-*H. pylori* regimens^38^ ^,^ ^373^.

Experimental studies have demonstrated that certain probiotics can modulate the gastric microbiota, secrete antibacterial substances, compete with *H. pylori* for adhesion to the gastric mucosa, and stimulate immune responses and mucin production^374^ ^,^ ^375^. Despite these findings, studies demonstrated that probiotics alone offer minimal eradication efficacy (averaging 14%) and are not indicated as monotherapy; their primary role is as an adjunct to classic antibiotic treatment^376^.

Several randomized clinical trials, meta-analyses, and systematic reviews have consistently demonstrated that adding probiotics to different anti-*H. pylori* regimens improves eradication rates (by 8% to 16%) and significantly reduces the incidence of side effects such as diarrhea, nausea, and abdominal pain^377^ ^-^ ^381^. Across multiple clinical studies, the reduction in adverse effects ranged from 30% to 59%^378^. These results are consistent across different populations and geographical regions^378^ ^,^ ^379^.

Favorable results were described when using specific strains (either single- or multi-strain formulations)^377^ ^-^ ^381^. Some authors noted more robust effects, both in therapeutic efficacy and side-effect reduction, with multi-strain probiotic regimens, especially those containing *Lactobacillus*, *Bifidobacterium*, and *Saccharomyces* ^377^ ^-^ ^381^. Some evidence suggests that higher doses and a longer duration of probiotic supplementation (>2 weeks) yield better outcomes^375^ ^,^ ^379^.

More recent studies have found even more favorable results, concluding that the adjunctive use of certain probiotic strains can enhance efficacy and improve tolerability across different *H. pylori* eradication regimens^379^ ^-^ ^381^. Notable among these is the study by Casas Deza et al., which presented data from the European Registry on *H. pylori* Management and provided the largest cohort analyzed to date (2013 to 2021) regarding the addition of probiotics to eradication regimens in clinical practice^379^. The authors highlighted the genera and strains linked to the highest therapeutic efficacy and identified which eradication regimens benefit most from the supplementation.

Despite these recent, highly favorable data, the topic remains controversial due to the vast heterogeneity in the strains and doses utilized, the timing (before and/or during and/or after antibiotics), and the duration of supplementation^5^ ^,^ ^38^ ^,^ ^51^. We conclude that probiotic supplementation is a plausible and highly promising adjunctive strategy. Specific probiotic strains can confer a beneficial effect during anti-*H. pylori* treatment, driving higher eradication rates and lowering antibiotic-induced side effects. However, it remains necessary to identify the ideal genera/strains and combinations to justify a strong recommendation for probiotic supplementation in *H. pylori* treatment^51^ ^,^ ^38^ ^,^ ^375^.

## Statement 34

## *H. pylori* eradication partially restores gastric microbiota diversity. However, the antibiotic therapy used for this purpose can cause transient or, rarely, persistent gut dysbiosis.

**Agreement: 91%**

**GRADE: 2B | Strength of recommendation: weak**

**Quality of evidence: moderate**

*H. pylori* infection undeniably modifies the gastric microbiota. This modification, however, is directly related to the specific bacterial strain, its aggressiveness, the characteristics of the host’s stomach, and the inflammatory response.

Classically, initial *H. pylori* infection drives a gastrin increase. This can induce hydrochloric acid hypersecretion among patients with a high parietal cell mass and low production of interleukin-1 beta and TNF-alpha (these cytokines are potent antisecretory agents). This patient group will experience a reduction in gastric microbiota diversity, driven by the strong bactericidal effect of HCl. Conversely, among patients with a reduced number of oxyntic cells and elevated production of the aforementioned cytokines, acid secretion undergoes little change and may even decrease over time due to eventual antral and fundic atrophy. In this scenario, the elevated pH promotes an increase in the number and diversity of the gastric microbiota. Gastric bacterial overgrowth is a known risk factor for neoplasm development^382^ ^,^ ^383^.

The presence of hypochlorhydria also ultimately impacts the intestinal microbiota, potentially fostering small intestinal bacterial overgrowth and its associated complications^384^.

*H. pylori* eradication can alter gastric secretory capacity due to the reasons outlined above, exacerbated by the antimicrobial effect of the medications used in eradication therapy. This acutely reduces bacterial diversity and richness, leading to changes in the composition of microbial communities, including a decrease in *Enterococcus* and an increase in *Lactobacillus*, *Bifidobacterium*, and *Bacteroides* ^385^. These alterations tend to be most pronounced in the first few weeks post-treatment, followed by a gradual recovery of microbial diversity and composition over months, generally returning to baseline patterns within 1 year^386^ ^,^ ^387^. Reports indicate minor changes in some genera that may persist in isolated cases^388^. The concomitant use of specific probiotic strains or combinations can attenuate antibiotic-induced dysbiosis, promoting a faster recovery of the intestinal microbiota^385^ ^,^ ^389^.

Beyond bacterial alterations, evidence suggests that *H. pylori* eradication also affects the intestinal virome, reducing viral diversity and altering phage-bacterium interactions-effects that may persist for up to 6 months, especially among patients who underwent multiple courses of antibiotics^390^ ^,^ ^391^.

## Data Availability

not applicable - the study did not use research data
