# ERRATUM: Vth Brazilian Consensus Conference on *Helicobacter pylori* infection

**DOI:** 10.1590/S0004-2803.24612026-E43

**Published:** 2026-10-05

**In article:** Coelho LGV, Sanches BSF, Maguilnik I, Marinho JR, Chaves SR, Ramos AFP, et al.Vth brazilian consensus conference on *helicobacter pylor*i infection. Arq gastroenterol. 2026;63:e26043. Doi: 10.1590/s0004-2803.24612026-043 

**Where it reads:**

 It is recommended that H2-receptor antagonists be discontinued **2 h** prior to diagnostic testing, PPIs and P-CABs 14 days prior, and antibiotics and bismuth compounds 4 weeks prior. Prior discontinuation of the use of sucralfate, alginate, and antacids is not required.

**It should read:**

 It is recommended that H2-receptor antagonists be discontinued **72h** prior to diagnostic testing, PPIs and P-CABs 14 days prior, and antibiotics and bismuth compounds 4 weeks prior. Prior discontinuation of the use of sucralfate, alginate, and antacids is not required.

